# Ocotillo optimization-driven deep learning for bone marrow cytology classification

**DOI:** 10.1371/journal.pone.0330228

**Published:** 2025-08-29

**Authors:** Doaa Sami Khafaga, El-Sayed M. El-kenawy, Faris H. Rizk, Marwa M. Eid, Ehsaneh Khodadadi, Nima Khodadadi

**Affiliations:** 1 Department of Computer Sciences, College of Computer and Information Sciences, Princess Nourah bint Abdulrahman University, Riyadh, Saudi Arabia; 2 Department of Programming, School of Information and Communications Technology (ICT), Bahrain Polytechnic, Isa Town, Bahrain; 3 Applied Science Research Center, Applied Science Private University, Amman, Jordan; 4 Department of Communications and Electronics, Delta Higher Institute of Engineering and Technology, Mansoura, Egypt; 5 Faculty of Artificial Intelligence, Delta University for Science and Technology, Mansoura, Egypt; 6 Jadara University Research Center, Jadara University, Irbid, Jordan; 7 Department of Chemistry and Biochemistry, University of Arkansas, Fayetteville, Arkansas, United States of America; 8 Department of Civil and Architectural Engineering, University of Miami, Coral Gables, Florida, United States of America; Duy Tan University: Dai Hoc Duy Tan, VIET NAM

## Abstract

Manual diagnosis of hematological cancers like leukemia through bone marrow smear analysis is labor-intensive, prone to errors, and highly dependent on expert knowledge. To overcome these limitations, this study introduces a comprehensive deep learning framework enhanced with the innovative bio-inspired Ocotillo Optimization Algorithm (OcOA), designed to improve the accuracy and efficiency of bone marrow cell classification. The contributions include developing a baseline Convolutional Neural Network (CNN) that achieves an initial accuracy of 86.29%, surpassing existing state-of-the-art deep learning models. Further integrate the binary variant of OcOA (bOcOA) for effective feature selection, which reduces the average classification error to 0.4237 and increases CNN accuracy to 93.48%. Additionally, utilize the continuous version of OcOA for hyperparameter optimization, further enhancing CNN performance to a maximum accuracy of 98.24%. Crucially, this optimization also results in a substantial clinical performance gain, with sensitivity increasing from 86.02% to 98.34% (+12.32%), specificity rising from 86.53% to 98.14% (+11.61%), and the false negative rate being significantly reduced, thereby enhancing diagnostic reliability in critical scenarios. These findings highlight the potential of metaheuristic optimization techniques to improve the effectiveness of deep learning models in clinical diagnostics quantifiably. The proposed approach demonstrates measurable gains in automated cytology technology, offering a scalable, interpretable, and accurate solution for hematological screening applications.

## 1 Introduction

To the extent that accurate classification of bone marrow cells establishes a key role in the diagnosis and monitoring of hematologic malignancies and disorders, such as different forms of leukemia, myelodysplastic syndromes, and aplastic anemia, a need for suitable morphological and histochemical typing of such cells has never been higher [1]. The bone marrow examination is an established clinical protocol used to assess the composition, morphology, and cellular health of hematopoietic tissue, obtained through biopsy or aspiration techniques [2]. As a critical diagnostic window, it provides information on blood cell production dynamics and pathological deviations that are not evident in peripheral blood examination alone. Due to the vast amount of diagnostic samples becoming too complex, too large, and too variable to optimize for cell classification, standardization and automation of bone marrow cytology, particularly given cell type classification, have emerged as pressing priorities within the clinical and research domains [3].

Over the past few years, large-scale and expertly annotated datasets have become available, accelerating a fundamental shift in machine learning (ML) and deep learning (DL) techniques in medical image analysis [4]. For instance, the Bone Marrow Cell Classification dataset comprises over 170,000 de-identified cell images from more than 945 patients, which is a notable example. Expert hematologists carefully annotated images and then processed them using high-resolution microscopy under constant staining conditions, as led by the Munich Leukemia Laboratory, utilizing the May—Grünwald—Giemsa/Pappenheim stain [5]. Image acquired with 40x magnification and oil immersion optics to capture morphological fidelity. The dataset represents a representative hematological diagnostic practice in a specialized clinical setting, encompassing a broad range of disease types and cellular morphologies. This type of resource has the potential to be an outstanding training ground for building trustworthy computational models that automate and enhance the cell classification process [6].

The integration of artificial intelligence (AI), specifically deep learning paradigms, into this diagnostic pipeline offers new opportunities to provide better support for making informed and quicker decisions about the disease, early disease detection, and workflow acceleration [7]. Modern neural networks, including Convolutional Neural Networks (CNNs) and Vision Transformers (ViTs) based models, can learn hierarchical and more abstract patterns, similar to how humans do, from raw data without human intervention. Their ability to capture complex, spatial, and textural patterns places them well to handle the morphological subtlety of bone marrow cytology. In addition, with the help of unlabeled data using self-supervised frameworks like SimCLR and MoCo, it makes sense to utilize unlabeled data to enhance generalization and reduce dependence on labor-intensive tasks. However, despite these advancements, several technical and methodological issues still hinder the creation of reliable, fully autonomous classification systems in this domain [8].

One of the main obstacles is that image-derived features are highly dimensional, primarily when deep networks derive representations across multiple levels of abstraction [9]. While rich in potential information, this dimensionality can suffer from the so-called ‘curse of dimensionality,’ making computations so expensive to be impractical and inducing feature redundancy [10]. Redundant or highly correlated features in training datasets can cause the training signal-to-noise ratio to decline and negatively impact classifier performance, while also increasing model complexity without a corresponding performance improvement. Moreover, deep learning models are susceptible to their hyperparameter configurations, which include learning rate schedules, regularization terms, architectural depths, and batch sizes. If the hyperparameters are not tuned properly, they will lead to suboptimal convergence behavior and poor generalization, especially when the dataset is imbalanced with a single class or when there are subtle variations between classes, as is often the case with many hematological disorders [11].

Then, overfitting is another problem, where models perform exceptionally well on training data but struggle to generalize to previously unseen samples. In particular, this is a problem when the cost of false negatives or false positives is clinically significant, such as in medical imaging tasks [12]. Further complicating the model’s generalization to different clinical scenarios is the variability introduced by patient demographics, sample preparation protocols, and staining artifacts. For this reason, many agree that any effective solution must rely upon both an intelligent feature selection to reduce the solution space dimensionality and rigorous hyperparameter optimization to stabilize the solution.

Based on the considerations above, this study aims to develop a comprehensive and data-driven framework for the automated classification of bone marrow cells. At the center of this framework, deploy multiple state-of-the-art deep learning models such as CNNs, ViTs, Generative Adversarial Networks (GAN), SimCLR, and MoCo architectures, which correspond to different paradigms of supervised and self-supervised learning [13]. They are also evaluated in terms of their ability to classify under different optimization schemes, as well as their classification efficacy. The study combines the most recent metaheuristic algorithms for feature selection to mitigate the impact of high-dimensional input features and enhance computational efficiency. Others include an adaptive binary form of nature-inspired optimizers designed to traverse the discrete search space of feature subsets with minimal computational overhead [14].

The study also emphasizes the use of continuous metaheuristic optimization for hyperparameter tuning to ensure that the learning algorithms operate in their optimal configurations, aligning with the intrinsic structure of the dataset. Adopting this holistic approach (bridging model architecture selection with metaheuristic-based optimization) aims to cleanly establish a high-performance classification pipeline that scales and is clinically practical.

This research examines and quantifies the benefits of combining deep learning architectures and bio-inspired metaheuristic techniques in the context of bone marrow cell classification. Overall, this research aims to accelerate the automation of hematological diagnostics, minimize the need for expert intervention, and ultimately improve the speed, accuracy, and accessibility of diagnostic services in medical institutions.

This work presents a new and unified deep learning framework that utilizes a new metaheuristic algorithm for the feature selection and the hyperparameter tuning stages of automated bone marrow cell classification. The main contributions of this study can be summed up as follows:

- The Ocotillo Optimization Algorithm (OcOA), a bio-inspired meta-heuristic optimization algorithm, is proposed. Both binary and continuous versions of OcOA are applied to discrete feature selection and continuous hyperparameter tuning problems within a unified optimization pipeline, respectively.
- An end-to-end classification architecture is developed by integrating deep learning models—such as Convolutional Neural Networks (CNNs), Vision Transformers (ViTs), and Generative Adversarial Networks (GANs), including SimCLR and MoCo—with metaheuristic-guided optimization, aiming at high-precision bone marrow cytology analysis.
- Data dimensionality reduction through the binary Ocotillo Optimization Algorithm (bOcOA), the retention of discriminative power, and improved generalization of the model are evaluated based on performance metrics derived from bOcOA-based feature selection.
- The CNN classifier is optimized by employing OcOA in its continuous form, resulting in enhanced accuracy and efficiency through hyperparameter tuning.
- A comprehensive comparative analysis is performed between the proposed framework based on the method (OcOA) and several state-of-the-art optimization algorithms, including Particle Swarm Optimization (PSO), Grey Wolf Optimizer (GWO), Firefly Algorithm (FA), Genetic Algorithm (GA), Whale Optimization Algorithm (WOA), as well as their binary variants.
- The versatility and robustness of the proposed optimization strategy are validated across multiple deep learning architectures, highlighting its adaptability to diverse learning paradigms, including supervised and self-supervised models.
- A reproducible, scalable, and clinically relevant solution for automated hematological image classification is established, demonstrating potential implications for diagnostic support within real-world laboratory workflows.

The rest of this paper is then organized as follows. The second section reviews comprehensive related work, including advancements in leukemia classification using deep learning and metaheuristic optimization techniques. Sect 3 introduces the Materials and Methods section, which includes the specifics of the dataset, the preprocessing pipeline used on it, the model architectures employed, and, finally, the proposed Ocotillo Optimization Algorithm (OcOA) for both feature selection and hyperparameter tuning. In Sect 4, empirical results include baseline model performance, feature selection comparison results, and optimization on classification accuracy. In the remaining Sect 5, the findings are discussed within the framework of previous research, the implications of the proposed method are interpreted, and the strengths and weaknesses of the methodological approach are examined. Sect 6 concludes the study by summarizing the contributions and providing suggestions for future work to improve automated hematological diagnostics.

## 2 Literature review

The malignant blood disease leukemia causes abnormalities in white blood cell production while accelerating at a fast pace, which is detrimental to patient outcomes without an early and correct diagnosis. Medical experts traditionally use microscopic examination of blood or bone marrow smears to diagnose leukemia. The manual analysis method enables experts to visually inspect cells for their basic appearance. Still, experts take too long to make different observations, and skilled personnel face resource constraints. Research and development of automated diagnostic systems based on artificial intelligence (AI) with machine learning (ML) and deep learning (DL) technologies have gained intense attention due to these systems’ constraints.

Recent developments in ML and DL technologies have enabled researchers to create robust diagnostic systems that analyze complex visual data with high accuracy levels. The diagnostic pipelines contain components that link extraction features with optimization and classification routines that researchers have intensively scrutinized for leukemia analysis. The review examines the advancements by organizing previous studies into groups focused on (1) performance-improving optimization methods, along with (2) dimensionality-reducing feature selection methods and (3) subtype disease prediction approaches.

### 2.1 Metaheuristic optimization in leukemia diagnosis

The diagnostic accuracy improves when natural and biological process-based optimization algorithms fine-tune feature subsets and classifier parameters. Researchers successfully applied WOA and SVM for detecting ALL through a method that resulted in 98.42% accuracy as reported in [15]. The evolutionary algorithm demonstrates how it efficiently searches hyperparameter spaces to enhance conventional classifier outcomes in medical datasets with complex characteristics.

Likewise, the k-means clustering for classifying all subtypes has been applied in conjunction with the Enhanced Grey Wolf Optimizer (EGWO) to achieve an accuracy of 99.22% [16]. These approaches demonstrate the success of nature-inspired optimization in addressing problems related to model architecture selection, reducing overfitting, and selecting salient features, particularly in high-dimensional medical imaging data.

### 2.2 Deep learning and hybrid architectures

However, Convolutional Neural Networks (CNNs) and other deep learning architectures have become the de facto choice for extracting hierarchical representations from the raw image inputs because they are automatic systems that can learn well. As demonstrated in work by [17], where the classifier and PSO are used, the CSO augmented model is built using a ResNet50 classifier, achieving a high accuracy of 99.84%. It is thus confirmed that the deep architectures complemented by metaheuristic tuning can potentially learn morphology patterns indicative of leukemia.

Another alternative study, combining GoogleNet with an 88-layer deep CNN by [18], utilized binary whale optimization and achieved 99.15% accuracy in all subtype classification. These results suggest that deep networks can learn detailed visual features and adjust their parameters to align with disease-specific discriminative cues.

This further supports the argument because the model proposed by [19] used a Deep Convolutional Neural Network (DCNN) with the addition of a Fuzzy Fusion Hunger Games Optimization (FFHHO) mechanism. The leukemia detection was achieved with a TPR of 91.7% and a TNR of 92.25%, indicating balanced sensitivity and specificity, two key metrics in the medical viability itself.

In addition, feature selection across several CNN architectures was optimized by a firefly algorithm, as shown in [20]. Results of the entropy-controlled firefly mechanism yielded impressive classification accuracies of more than 99%, demonstrating the effectiveness of the hybrid evolutionary method in creating a CNN-based diagnostic system.

### 2.3 Expanded classification strategies and model integration

In addition to leukemia, several other hematological malignancies have been classified with AI-based classification frameworks beyond leukemia. In [21], a hybrid CNN-ANN model was used to determine a 100% accuracy in identifying Multiple Myeloma, a cancer of plasma cells. This demonstrates the use of such layered neural network architectures to detect subtle variations in the related pathologies.

In this paper, we have applied a dense neural network (DNN) combined with DenseNet201 features extracted via a DenseNet201-based feature extractor and optimized using HHO to reach 99.33% classification accuracy for the lymphoma subtypes. The success of dense architectures and the effective use of features in deciphering the complex yet rich histological signature of cancer cells illustrates this.

Presents another novel approach [22] through a Hybrid CNN model coupled with an Interactive Autodidactic School (HCNN-IAS) learning strategy, for which they achieve a classification of 99%, validating the effectiveness of integrating automated self-learning schemes in the regular deep learning pipeline.

Election-Based Chameleon Swarm Algorithm (E-CSA) is introduced by [23] for the early 183 stage leukemia detection using the MTResUnet3+ segmentation model. The dual layering of this framework combines semantic segmentation and optimization to make better precision in region localization and classification. Like CoTCoNet, proposed in [24], a model that combines convolutionality and attentionality in feature extraction, the CoTCoNet model achieved 98.94% accuracy across different leukemia datasets with attention-based learning in the medical space.

In [25], segmentation and classification were performed with an African Buffalo and African Vulture Optimization (AB-AVO) based on RNNs. Therefore, hybrid evolutionary algorithms and temporal learning structures yielded nearly perfect diagnostic outcomes, achieving 100% accuracy in classifying all IDB datasets.

The last is a comprehensive review from [26] of many different ML strategies used to classify leukemia, establishing a foundation for diverse approaches’ evolution and performance comparison. Finally, the study presents what is becoming a trend of increasing automation, algorithmic interpretability, and multi-stage optimization in medical diagnostics.

### 2.4 Summary and research gap

Table 1 illustrates that current studies have achieved significant results in leukemia classification using AI-driven techniques, including CNN and various metaheuristic algorithms. Many of these efforts have yielded high diagnostic accuracies exceeding 99%. However, there are several significant constraints that need to be overcome before the further development of more unified and scalable diagnostic frameworks can proceed.

**Table 1:** Summary of literature review.

Reference	Objective	Methodology	Key Findings
[15]	Automated ALL detection	WOA-SVM, feature selection	98.42% accuracy
[16]	ALL subtype classification	EGWO, k-means clustering	99.22% accuracy
[17]	Multi-class leukemia classification	ResNet50, PSO, CSO, LR	99.84% accuracy
[18]	Binary and subtype classification of ALL	GoogleNet, deep CNN, whale optimization	99.15% accuracy
[19]	Leukemia detection	FFHHO-based DCNN	91.7% TPR, 92.25% TNR
[20]	Leukemia classification across multiple datasets	Multiple CNNs, firefly optimization	Accuracy up to 99.64%
[21]	Multiple Myeloma detection	CNN-ANN hybrid model	100% accuracy
[27]	Lymphoma subtype classification	DenseNet201, HHO	99.33% accuracy
[22]	Hybrid leukemia classification	HCNN-IAS model	99% classification accuracy
[23]	Early-stage leukemia detection	MTResUnet3+, E-CSA, MAA-DCNN	Enhanced classification accuracy
[24]	Leukemia classification framework	CoTCoNet, transformer-CNN	98.94% accuracy
[25]	ALL segmentation and detection	AB-AVO, RNN	100% accuracy
[26]	Overview of ML in leukemia classification	Various ML techniques	Analysis of classification trends

First, most prior work has regarded feature selection and hyperparameter optimization as individual and sequential processes employing different algorithms for each task. Over time, real phenomena create fragmentation, resulting in inconsistent optimization outcomes, increased complexity, and decreased efficiency. Furthermore, although each binary and continuous metaheuristic has demonstrated strong results, few studies have created a single algorithm that handles both discrete and continuous search spaces within a unified structure.

Secondly, a coherent pipeline doesn’t exist that features selection, hyperparameter tuning, and training in any way. However, many reported models optimize individual components in isolation, severely restricting the synergy obtained by jointly optimizing.

The present study introduces the Ocotillo Optimization Algorithm (OcOA) and its binary variant (bOcOA) as a unified solution of feature selection and hyperparameter tuning for a generic standard form of the original HOSSE data. In contrast to independent algorithms as in existing works, the approach uses a consistent metaheuristic framework to navigate both domains. It enables a streamlined optimization process, simplifies model generalization, and reduces the complexity of developing the proposed strategy.

At the same time, when feature selection and parameter tuning are integrated into a coordinated pipeline, the results show that they can improve diagnostic accuracy and computational efficiency, thereby enhancing the performance of multiple deep learning models, such as CNN, ViT, GAN, SimCLR, and MoCo. Overall, it bridges the gap between the optimization stage and classification methodological divide with a replicable and adaptable framework for high-precision, image-based leukemia diagnosis.

## 3 Materials and methods

**Fig 1:**
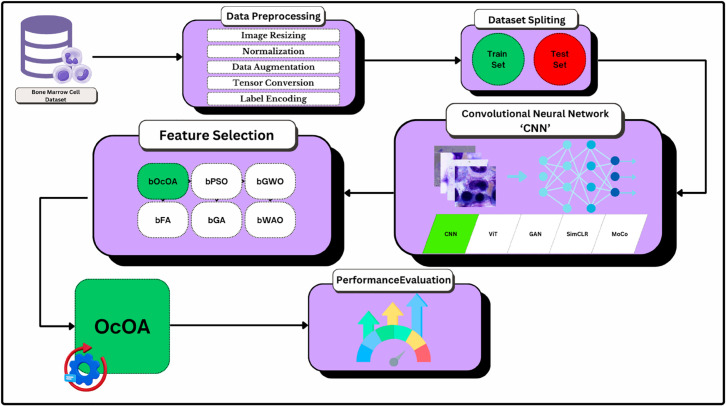
Overview of the proposed deep learning and feature selection framework for bone marrow cell classification.

The ability to classify medical images has historically had a lower score due to the lack of advancement in deep learning and feature selection techniques. Still, today, this is not the case due to these advancements. A high degree of importance is given to bone marrow cell analysis to diagnose various hematological conditions. This study employs an overall framework that integrates multiple essential steps, including data preprocessing, dataset splitting using CNNs, feature selection, and performance evaluation. Robust and interpretable predictions are achieved through a hybrid approach that combines convolutional architectures with bio-inspired optimization algorithms. Fig 1 illustrates the comprehensive workflow of the proposed methodology (i) by which the proposed processing pipeline for raw data to performance assessment is shown step by step.

To facilitate reproducibility and clarity, we detail all stages of the pipeline in full, including preprocessing, model architecture, training protocols, and optimization techniques. The framework is implemented in Python using well-established libraries, including TensorFlow/Keras for deep learning, Scikit-learn for metric evaluation, and NumPy for data manipulation. Metaheuristic optimization algorithms were implemented from first principles based on their mathematical definitions, with parameter settings and evaluation procedures provided in the corresponding sections.

The dataset was partitioned using a stratified 70:30 train-test split, ensuring balanced class representation across both subsets. Each model was trained for 50 epochs with a batch size of 32. The loss function used for multi-class classification was categorical cross-entropy, suitable for softmax-activated output layers in CNNs. The optimizer used in the initial training phase was Adam, chosen for its adaptive learning rate capabilities, with a fixed learning rate of 0.01.

Dropout was applied at a rate of 0.3 after dense layers to mitigate overfitting. Model weights were initialized using the He-normal initialization, and early stopping was monitored based on the validation loss with a patience of 5 epochs. The input images were resized to a consistent resolution, normalized to the [0, 1] range, and augmented using random flips and rotations. All training runs were conducted on an NVIDIA-enabled GPU workstation, and random seeds were fixed globally to ensure deterministic reproducibility. These settings were used consistently across models unless otherwise specified during metaheuristic-based tuning.

### 3.1 Dataset

#### 3.1.1 Dataset description.

This study employed a part of the Bone Marrow Cell Classification dataset [28]. This large, carefully crafted biomedical image corpus can simulate the complexities and heterogeneities inherent to hematological diagnostics. A total of 171,375 high-resolution, de-identified images of cellular images derived from bone marrow smears from 945 individual patients form the content of this dataset. Numerous hematopathologists and cytology experts from the Munich Leukemia Laboratory (MLL), an internationally renowned diagnostic and research institute specializing in leukemic and marrow-related disorders, expertly annotate each image.

Given that acquisition protocols conformed to stringently applied standards of morphological uniformity and diagnostic importance, the addition of FIJ samples is not expected to significantly influence overall reliability or impact any results. All smears were stained using a well-established differential staining method, the May-Grünwald-Giemsa/Pappenheim protocol. The dual stain provides comparable contrast for different cellular structures, enabling the clear separation of precursor cells, blasts, erythroid lineage cells, granulocytes, lymphocytes, and other cell types, thus facilitating functional differential diagnosis.

The brightfield microscope was operated at a magnification factor of 40x and oil immersion optics to retain high-resolution morphological detail for image acquisition. There are also reasons this setup was chosen: it can offer a consistent depth of field and color fidelity, which are two key factors in achieving well-defined results within the limits of cytological imaging. A further set of image scanning and digitization procedures was completed using proprietary image scanning equipment designed and developed at Fraunhofer IIS, along with associated post-processing pipelines tailored for Helmholtz Munich to ensure annotation integrity and consistent resolution, lighting, and focus.

The final dataset encompasses a broad diagnostic spectrum, including acute myeloid leukemia (AML), acute lymphoblastic leukemia (ALL), chronic myeloproliferative neoplasms (MPN), myelodysplastic syndromes (MDS), plasma cell disorders, as well as other marrow abnormalities. This diversity makes the dataset a realistic surrogate of the diagnostic workload of large-scale hematology laboratories.

The dataset contains structured metadata for each image (class label to type of bone marrow cell), and all images are also provided in their respective classes. Finally, these are the target variables for the supervised learning tasks. In a multi-class classification framework, these class labels were used for this study, wherein each instance belongs to one and only one of the predefined cellular categories.

To preserve the statistical validity and reproducibility of the experiments, the dataset was stratified and sampled into training, validation, and testing splits. With this strategy, the class distribution is further preserved on all three subsets proportionally, rather than through class imbalance mitigation, so that the entire data distribution is still represented by training and evaluating the models. The split ratio was set empirically to 70% for training, 15% for validation, and 15% for testing, respectively. Then, the images were randomly shuffled, ensuring that all images for partitioning were free from any ordering bias, which helps ensure that the model evaluation results are robust.

**Fig 2:**
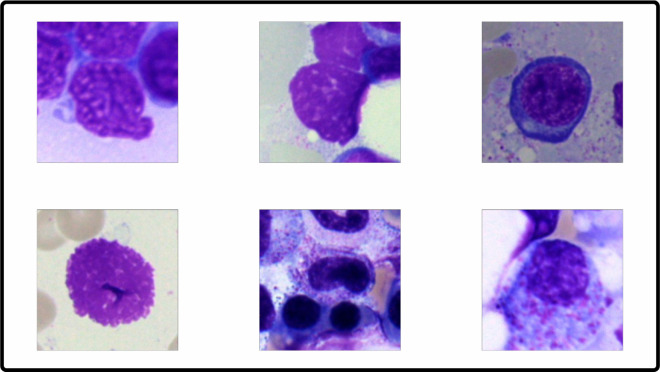
A sample image from the dataset used in this study.

To better understand the data being used, Fig 2 shows a sample image from the dataset. This visualization illustrates the format and structure of the data before further analysis or processing.

#### 3.1.2 Data preprocessing.

Given the dataset’s image-based nature, as most of the Bone Marrow Cell Classification dataset consists of images, a comprehensive data preprocessing was performed to feed the images to the deep learning architectures. It was designed to standardize the samples for uniformity, enhance the signal-noise ratio, and accommodate convolutional and transformer-based models.

First, all raw images were resized to the exact spatial resolution of 224×224 pixels [29]. This resolution was chosen to match the input dimensionality requirements of standard deep convolutional networks, such as Vision Transformers (ViTs), and to preserve morphological fidelity in classifying hematological images. Bilinear interpolation was used for resizing operations to preserve edge integrity and avoid aliasing artifacts [30].

Pixels were then normalized within each image based on pixel intensity values. The RGB colorspaces were converted to floating point and scaled to the same range [0,1] for each image. Moreover, global z-score normalization was performed by subtracting the dataset-wide mean pixel intensity and dividing by the standard deviation such that all input features have a zero mean and unit variance [31]. This step is crucial to speed up convergence and stability during training in gradient-based optimization algorithms.

To encourage a higher level of generalization of the model and avoid the problem of overfitting, a set of data augmentation techniques was only applied to the training set. These augmentations included random rotations ($\pm 15^{\circ }$), vertical and horizontal flipping, color jittering (changes in brightness, contrast, and saturation), random cropping, and the addition of Gaussian noise. The augmentation was probabilistically applied during training, so no two epochs saw augmentation of the same augmented view of an image. The model can learn invariant features under simulated real-world variations observed in clinical microscopy, thereby facilitating the search and identification of models that have learned these invariant features.

After encoding images into numerical tensors, they were batched using an efficient data loader that preprocesses images in parallel, thereby maximizing GPU utilization during training [32]. One hot-encoded label was used to ensure the same number of vectors matched the softmax output layer of the classification models.

Finally, the preprocessing phase was designed to produce a high-quality, standardized, and augmented image corpus in a format suitable for feeding into state-of-the-art deep learning pipelines. In the context of feature selection, model training, and hyperparameter optimization procedures, it serves as a base for these later steps.

### 3.2 Machine learning models

Utilize diverse deep-learning architectures to address the bone marrow cell classification task. To capture a wide range of modern visual representation learning paradigms, we chose these models to correspond to a broad spectrum of both supervised and self-supervised learning strategies. In particular, we implemented Convolutional Neural Networks (CNNs), Vision Transformers (ViTs), Generative Adversarial Networks (GANs), and two types of contrastive learning frameworks: Simple Contrastive Learning of Representations (SimCLR) and Momentum Contrast (MoCo). Due to their distinctive inductive biases and distinct training methods, each architecture provides a valuable basis for comparative evaluation and ensemble integration in hematological image analysis.

In this research, the Convolutional Neural Network (CNN) [33] is employed, a foundational model used as a baseline to assess other architectures. CNNs have been proven to be highly validated in medical image classification tasks, mainly due to their intrinsic capabilities for local feature extraction, hierarchical representation learning, and translational invariance. The network architecture is based on a standard deep convolutional lineup of consecutive convolutional layers with ReLU activations, max-pooling layers for spatial downsampling, and fully connected output layers, resulting in a softmax output. This structure is very suitable for capturing the morphological characteristics of bone marrow cells, including nuclear size, cytoplasmic granularity, and contour characteristics, which are necessary for differential diagnosis in hematopathology.

The Vision Transformer (ViT) is a model that helps with image understanding tasks by departing from convolutional priors and reinforcing the transformer architecture (designed for natural language processing tasks) for image understanding tasks [34]. While CNNs use overlapping image patches that are flattened and embedded linearly into the CNN layers, and then processed with multiple layers of multi-head self-attention feed-forward networks, ViTs operate on non-overlapping patches, processed with multi-head self-attention and feed-forward networks. In the context of histopathological analysis of cell images, diagonal global contextual relationships are meaningfully distributed over various spatial regions of the cell image, a pattern that the ViT mainly handles well. Moreover, the model’s reliance on attention mechanisms facilitates interpretability, allowing for insight into the decision-making process through attention maps.

A Generative Adversarial Network (GAN) was introduced to incorporate architectural diversity into the modeling pipeline. GANs contain two neural networks, the generator and the discriminator, which are trained together in an adversarial manner. The discriminator is tasked with distinguishing real from synthetic images, whereas the generator aims to produce images that cannot be distinguished from the training data [35]. In this case, while GANs are usually employed for image synthesis, a classification-augmented GAN architecture was utilized, where the discriminator not only classifies the images as fake or real but also provides class probabilities. Providing dual adaptation signals for adversarial training and cell classification enables a discriminator to obtain training benefits while learning discriminative features for cell classification. GANs are especially favorable when synthetic data generation can aid in balancing classes and improving generalization in datasets.

In this respect, two self-supervised learning (SSL) models (SimCLR and MoCo) were used to learn feature representation using unlabeled data without direct supervision [36]. The SimCLR is based on the concept of contrastive learning, where the model must distinguish between augmented views of the same image within the same latent space, bringing those views closer together, and opinions of different images further apart. Following a ResNet-based encoder, the architecture follows with a projection head that projects the representations to a contrastive loss space. It can learn the invariant features from the augmented pairs and then fine-tune them on the downstream classification task using a small, labeled subset of the dataset.

Momentum Contrast (MoCo) employs a dynamic dictionary mechanism for negative sampling, utilizing a momentum-updated encoder in another contrastive learning paradigm [37]. In contrast, MoCo approaches the same task using much smaller computational resources than SimCLR by exploiting a queue of encoded features. This approach facilitates the performance of a contrastive learning process in memory, which is particularly valuable for high-resolution medical image datasets. With the flexibility of MoCo in learning discriminative features from unlabeled data, it is an ideal component of the modeling strategy.

Overall, these five models (CNN, ViT, GAN, SimCLR, and MoCo) provide a rich landscape for comparing the two categories of bone marrow cell classification approaches: supervised and self-supervised. Due to their unique architectural designs and learning paradigms, they open the door for a detailed study of the role of different feature extraction and representation learning schemes on classification performance in complex biomedical imaging problems. Both methodological rigor and clinical relevance are ensured through the use of these models, given their state-of-the-art status in the computer vision literature and proven utility in similar medical imaging problems.

### 3.3 Metaheuristic algorithms

Metaheuristic algorithms have been successfully integrated into machine learning workflows in high-dimensional input spaces with non-convex optimization landscapes. Researchers have increasingly begun to utilize these algorithms across various scientific domains. Metaheuristics offer an alternative to existing feature selection and hyperparameter tuning strategies in the context of medical image analysis, particularly in the field of bone marrow cytology. The rigidity of a heuristic typically restricts such methods, often leading to the exhaustion of an exhaustive search or the use of domain-specific thresholds. Metaheuristic algorithms mimic natural phenomena, such as swarming behavior, evolutionary competition, or gravitational attraction, to enable an efficient search through a vast search space. This study systematically applies both the binary and continuous versions of metaheuristics to address two critical issues in deep learning-based classification: feature selection that is insensitive to dimensionality via dimensionality reduction and performance maximization that is insensitive to hyperparameters within deep learning.

#### 3.3.1 Role of metaheuristics in feature selection.

In any dimensional single-task classification problem, feature selection is a crucial part of the pipeline, as it helps increase model interpretability, reduces computational overhead, and mitigates the risks of overfitting. However, image-based classification, particularly when utilizing deep feature embeddings due to convolutional or transformer networks, yields a feature space that is usually very rich with redundant or irrelevant descriptors. Filter methods based on statistical correlation, greedy search wrapper, and embedded methods integrated within specific classifiers are traditional feature selection techniques that are often rigid, scalable, and model-dependent.

Utilize a set of binary metaheuristic algorithms to address these drawbacks, with the binary feature selection problem reformulated as a binary optimization problem. As represented, each candidate solution in this formulation, a binary string whereby each bit represents inclusion (1) or exclusion (0) of the corresponding feature, is described as a problem in the binary knapsack domain. The goal is to find the best subset of features such that the fitness function best reflects accuracy and the number of features.

A few binary metaheuristics used in this study are bFA, bGA, bWOA, bGWO, bPSO, and bOcOA, as listed in the abbreviation reference file. These are inherently population-based algorithms, as they iterate through operations on a set of candidate feature subsets, which are iteratively useful based on biological or physical processes. Thanks to this advantage in escaping local optima and dynamically adjusting the trade-off between exploration and exploitation, feature subset discovery amongst compact sets of features is made possible.

All metaheuristics are updated according to a well-defined update mechanism. For example, bPSO updates particle velocity and position in a binary feature space, while bGA represents it as a form of crossover and mutation in evolving solutions in different generations. Both the bGWO model’s socio-hierarchy and the hunting behavior of grey wolves, as well as the bWOA, attempt to mimic the bubble net feeding strategy of whales. In particular, the recently introduced bOcOA solves for performance in swarm dynamics inspired by cockroach social interactions and converges rapidly, adapting readily. This algorithm is run across multiple experimental runs to analyze its consistency, robustness, and capacity to reduce feature dimensionality without compromising classification accuracy.

#### 3.3.2 Role of metaheuristics in hyperparameter optimization.

Hyperparameter optimization is another critical component parallel to feature selection during training high-notch, deep-learning models. The behavior of the model, including its convergence stability and generalization capacity, depends on the hyperparameters. These two parameters are labor-intensive, time-consuming, and susceptible to subjective bias and entrapment in local optima. Among others (Such as Grid and random search), these methods are often used but become computationally prohibitive as a function of increasingly complex model architectures and large parameter spaces.

This study then applies continuous metaheuristic algorithms, such as automated optimizers, during hyperparameter selection because these challenges require addressing. In contrast to their binary counterparts used in feature selection, these continuous variants search in the real-valued space of candidate solution values, which represent a vector of hyperparameter values. Each solution is scored based on how well the model performs on a given validation set, using metrics such as accuracy, F1 score, or minimization of the loss function.

All of these are calibrated to solve the search space, which is also utilized, i.e., the continuous nature of the search space and the nature-inspired algorithms of the same family, namely OcOA, PSO, GWO, FA, GA, and WOA. For example, PSO stores particle positions and velocities as floating-point vectors, GWO represents leadership-based encircling behavior on continuous domains, and FA is an attractiveness-based model that incorporates distance and intensity-based moves. In particular, the OcOA is deployed due to its robust convergence behavior and low computational complexity, making it a suitable choice for deep learning optimization.

In these algorithms, hyperparameter configurations are initialized from diverse populations and evolved through subsequent iterations toward optimal regions of the search space. An optimization procedure for configuring the best-performing configuration(s) is repeated multiple times to ensure statistical robustness; the best-performing configurations across all validation folds are selected. The streamlined tuning process accelerates model development, enhancing predictive reliability and training efficiency, particularly in clinical applications where reproducibility and diagnostic precision are crucial.

In this study, metaheuristic algorithms address two primary challenges in biomedical image classification: dimensionality reduction and model optimization, utilizing adaptive, scalable, and biologically inspired search strategies. Indeed, their integration into the learning pipeline is expected to lead to compact models (combining accuracy and interpretability while remaining computationally economical).

#### 3.3.3 Proposed metaheuristic optimization and feature selection algorithm.

This study proposes to utilize one of the recently developed metaheuristic algorithms inspired by nature, called the Ocotillo Optimization Algorithm (OcOA), as the primary optimization engine for feature selection and hyperparameter tuning in the classification of bone marrow cells. OcOA is emulated from the adaptive behaviors of desert flora, specifically the ocotillo plant (Fouquieria splendens), widely known for its growth and blooming patterns in arid and shifting conditions. The algorithm was first presented to address essential issues in the theory of optimization, such as premature convergence, insufficient exploration, and poor adaptability in high-dimensional search spaces.

OcOA is an exploration and exploitation algorithm that leverages a dual-phase architecture, where stochastic variables and search behaviors control both the exploration phase and exploitation phase are strategically altered between each other (dynamically) in the real world [38]. During the exploration phase, the search space is filled with a wide distribution of candidate solutions using sinusoidal and cosine perturbations. The behavior of these perturbations is shaped by an amplitude factor (*A*), a distance function (*D*), and a feedback-informed learning rate (*L*), which are modulated over time to adapt the sense of convergence state of the algorithm.

As soon as promising regions of the search space can be identified, the OcOA enters the exploitation phase, where local refinements occur using Gaussian-based mutations, convergence factors, and non-linear scaling of previous search attempts. The adaptive feedback mechanism further regulates the convergence trajectory to balance the search between its global and local optima. Most importantly, the algorithm incorporates a convergence-aware control function (*K*) and a dynamic update rule for the learning factor (*L*). This allows the exploitation strategy to remain flexible and dynamic, responding to changes in fitness.

Here, we clarify key parameters used throughout the algorithm:

- *r*_1_, *r*_2_, *r*_3_ are uniformly distributed random numbers in [0,1] that introduce stochasticity into the update rules, helping to maintain diversity in the population and prevent premature convergence.
- Train refers to the current iteration number or a normalization scalar based on the training size, used to scale certain dynamic behaviors as the algorithm progresses.
- *K* is a convergence control factor computed adaptively to guide exploitation. It adjusts mutation strength based on convergence rate, helping OcOA shift from exploration to exploitation.

In the context of the feature selection task, the binary variant of the OcOA, namely bOcOA, is employed. This is the binarized version of OcOA, where candidate solutions are represented by binary strings, with each bit of a string encoding the presence or absence of a specific feature. OcOA can thus perform the combinatorial space search of feature subsets under the constraint that the resulting subsets are as compact as possible while maintaining high predictive accuracy. bOcOA maintains the core behavioral dynamics of the continuous bOcOA by projecting updates to bOcOA through a probabilistic sigmoid transformation that enforces binary constraints.

For hyperparameter optimization, OcOA was taken in the continuous form. The hyperparameters—learning rate, dropout ratio, number of hidden layers, and batch size—were represented in a constant vector form and optimized based on classification accuracy against a validation set. It progressively explored the hyperparameter space without resorting to local optima. It identified configurations that best aided ROCO in converging quickly and generalizing well across all the deep learning models used.

Finally, as presented in Algorithm 1, outline the complete procedural steps of the continuous OcOA algorithm, namely, the computation of sinusoidal amplitude adjustments, feedback-driven learning factors, and multi-stage Gaussian mutations. Together, these operators improve the search’s flexibility and robustness in nonconvex optimization landscapes with moderate complexity.

**Algorithm 1. Ocotillo Optimization Algorithm (OcOA) for Hyperparameter Optimization.**

1: **Initialize** population size *N*, maximum iterations *Maxiter*

2: **Initialize** random positions $\Theta_{i}$ for i=1,2,...,N

3: **Define constants:** $A,L,D,r_{1},r_{2},r_{3}$

4: **Set initial best solution:** $\Theta_{\text{best}}$

5: **for** *t* = 1 to *Maxiter* **do**

6:   **for** each solution $\Theta_{i}$ **do**

7:     Compute distance factor:


$$D=\frac{2\cdot \cos(\Theta )}{(\text{Train}{)}^{2}}$$

8:     Compute amplitude:


$$A=\sin\left(\frac{a}{b}\cdot \Theta \right)$$

9:     Compute learning factor:


$$L=\frac{1-2\sin(\Theta )}{(1+8\cos(\Theta ){)}^{2}}$$

10:     **Update position (Exploration):**


$$\Theta_{i}(t+1)=\Theta_{i}+A\cdot D+r_{1}\cdot L$$

11:   **end for**

12:   **Evaluate fitness** of all $\Theta_{i}(t+1)$

13:   **Update** $\Theta_{\text{best}}$ if better solution found

14:   **for** each solution $\Theta_{i}$ **do**

15:     Compute Gaussian mutation:


$$\Theta_{i}(t+1)=\text{Gaussian}(\mu )+L_{1}\cdot r_{1}\cdot \frac{A}{i\cdot \text{Train}}+\frac{F\cdot A}{o}$$

16:     Compute:


$$F=\sum_{n=0}^{10}\frac{\sin(n\cdot \Theta )}{(\text{Train}{)}^{2}}$$

17:     Update position (Alternative Mutation):


$$\Theta_{i}(t+1)=(r_{2}\cdot i^{2})\cdot {\left(\frac{K\cdot A}{(i\cdot \text{Train}{)}^{2}}\right)}^{2}$$

18:   **end for**

19:   **Update population** with new solutions $\Theta_{i}(t+1)$

20:   **Update** $\Theta_{\text{best}}$ if necessary

21:   **if** stopping criteria met **then**

22:     **Break**

23:   **end if**

24: **end for**

25: **Return** best solution $\Theta_{\text{best}}$

At the same time, bOcOA is used to solve the discrete optimization problem of determining the optimal feature subsets. The binary representation of each candidate solution consists of bits of length *d*; a bit value of 1 indicates the inclusion (1) of that corresponding feature, and a bit value of 0 means nothing was included or excluded (0). The algorithm adapts the exact overarching two-phase search overall algorithm by adjusting the update rules to suit binary encoding.

The algorithm first uses a sigmoid transfer function, followed by stochastic binarization to transition from real-valued exploratory to binary feature selection. Using this method, real-valued outputs are converted into binary states at probabilistic thresholds, allowing for the effective traversal of a discrete space of possible feature combinations.

The full binary OcOA procedure for feature selection is described in Algorithm 2.

**Algorithm 2. Binary Ocotillo Optimization Algorithm (bOcOA) for Feature Selection.**

1: **Input:** Feature dataset *X*, labels *Y*, population size *N*, max iterations *Maxiter*

2: **Output:** Best binary feature subset $\Theta_{\text{best}}$

3: Initialize binary population $\Theta_{i}\in \{0,1{\}}^{d}$ for i=1,2,...,N

4: Define constants and parameters: $r_{1},r_{2},r_{3},A,D,L$

5: Evaluate fitness for each solution $\Theta_{i}$ using a classifier (e.g., KNN)

6: Set best solution $\Theta_{\text{best}}$

7: **for** *t* = 1 to *Maxiter* **do**

8:   **for** each solution $\Theta_{i}$ **do**

9:     Compute:


$$D=\frac{2\cdot \cos(\Theta_{i})}{(\text{Train}{)}^{2}}$$

$$A=\sin\left(\frac{a}{b}\cdot \Theta_{i}\right),\;L=\frac{1-2\sin(\Theta_{i})}{(1+8\cos(\Theta_{i}){)}^{2}}$$

10:     Compute position update (exploration phase):


$$\Theta_{i}^{\prime }=\Theta_{i}+A\cdot D+r_{1}\cdot L$$

11:     Apply Sigmoid transfer function:


$$S(\Theta_{i}^{\prime })=\frac{1}{1+e^{-\Theta_{i}^{\prime }}}$$

12:     Binarize:


$$\Theta_{i}(t+1{)}^{j}=\left\{ \begin{array}{cc} 1 & \text{if }S(\Theta_{i}^{\prime }{)}^{j}>rand(),\\ 0 & \text{otherwise} \end{array}\right.$$

13:   **end for**

14:   Evaluate fitness of all $\Theta_{i}(t+1)$

15:   Update $\Theta_{\text{best}}$ if better solution found

16:   **for** each solution $\Theta_{i}$ **do**

17:     Exploitation phase:


$$\Theta_{i}^{\prime }=\text{Gaussian}(\mu )+L_{1}\cdot r_{1}\cdot \frac{A}{i\cdot \text{Train}}+\frac{F\cdot A}{o}$$

$$F=\sum_{n=0}^{10}\frac{\sin(n\cdot \Theta_{i})}{(\text{Train}{)}^{2}}$$

$${\Theta_{i}^{\prime }}^{\prime }=(r_{2}\cdot i^{2})\cdot {\left(\frac{K\cdot A}{(i\cdot \text{Train}{)}^{2}}\right)}^{2}$$

18:     Apply sigmoid and binarization as above to ${\Theta_{i}^{\prime }}^{\prime }$

19:   **end for**

20:   Replace old solutions with new binary solutions

21:   Update $\Theta_{\text{best}}$ if necessary

22:   **if** stopping criteria met **then**

23:     **Break**

24:   **end if**

25: **end for**

26: **return** $\Theta_{\text{best}}$

Chained together, these dual modes provide OcOA with a binary encoding for feature subset selection and a continuous encoding for hyperparameter tuning, thereby placing OcOA as a unified, adaptive, and high-performance optimizer. This integrated formulation, with clearly defined stochastic and convergence factors, ensures both global exploration and local exploitation are robustly executed across varying search domains. This represents a significant methodological advancement introduced into this study, enabling efficient operation in both discrete and continuous search domains through a single, biologically inspired framework.

#### 3.3.4 State-of-the-art metaheuristic optimization algorithms for comparative analysis.

To evaluate the effectiveness and efficiency of the proposed Ocotillo Optimization Algorithm (OcOA), it has been compared to five widely accepted metaheuristic algorithms that represent different biological or physical inspirations. It has proven to be an optimization algorithm of choice in optimization literature.

The comparative algorithms include:

- **Particle Swarm Optimization (PSO)** — PSO mimics the behavior of a swarm of particles moving within the search space [39], adjusting their position as they do so and infusing a few’s job experience with third-party experience in the collective.
- **Grey Wolf Optimizer (GWO)** — GWO, similar to the hunting behavior and leadership hierarchy in grey wolves in nature [40], utilizes the encircling and attacking mechanism to search and explore the search space. They are guided by alpha, beta, and delta wolves.
- **Firefly Algorithm (FA)** — Based on the bioluminescent communication of fireflies [41], FA employs attractiveness and distance-based movement to cluster around high-quality solutions in the fitness landscape.
- **Genetic Algorithm (GA)** — A Genetic Algorithm emulates the Darwinian process of natural selection with operators like selection [42], crossover, and mutation that guide solutions to evolve the next generation.
- **Whale Optimization Algorithm (WOA)** — It is inspired by humpback whales’ bubble net hunting strategy by integrating exploration and exploitation in spiral updating and encircling techniques [43].

The feature selection was performed using the algorithms mentioned above in their binary variants, namely bPSO, bGWO, bFA, bGA, and bWOA. Since subsets of features give candidate solutions in a complete feature set, each algorithm was adapted to operate in a binary solution space. The problem was to minimize classification error and to reduce the dimensionality of the input feature space.

They specifically used their *continuous versions* of their problem for **hyperparameter optimization**, i.e., PSO, GWO, FA, GA, WOA. In particular, these candidate solutions were vectors of continuous-valued hyperparameters in these scenarios. Aimed to find hyperparameter configurations that result in maximum classification performance based on cross-validated held-out metrics on a separate validation set.

The proposed OcOA framework is evaluated in terms of convergence speed, solution quality, computational efficiency, and robustness using these comparative algorithms as benchmarks. Including them makes it possible to ground the empirical performance of OcOA into careful and objective methodological baselines for both discrete and continuous optimization tasks.

### 3.4 Evaluation metrics

Several evaluation metrics were employed to rigorously assess the performance of the classification models and the effectiveness of the metaheuristic feature selection strategies used in this study. The selection of these metrics, based on aspects of classification efficacy and optimization robustness, includes predictive accuracy, error reduction, model generalization, and subset stability. The following subsections delineate the metrics used in the evaluation process of the deep learning models and the feature selection algorithms.

#### 3.4.1 Machine learning prediction metrics.

As shown in Table 2, we adopted six standard performance indicators for classifying tasks, including accuracy, sensitivity (true positive rate), specificity (true negative rate), positive predictive value (PPV), negative predictive value (NPV), and F1 score. The confusion matrix is the derivation from which these metrics are obtained. The predictions are based on false positives (FP), false negatives (FN), true positives (TP), and true negatives (TN). Combined, they provide a holistic view of how the classifier performs in terms of correctness, class discrimination, and its balance of precision and recall.

**Table 2:** Classification evaluation metrics and their mathematical definitions.

Metric	Mathematical Definition
Accuracy	$\frac{TP+TN}{TP+TN+FP+FN}$
Sensitivity (TPR)	$\frac{TP}{TP+FN}$
Specificity (TNR)	$\frac{TN}{TN+FP}$
Positive Predictive Value (PPV)	$\frac{TP}{TP+FP}$
Negative Predictive Value (NPV)	$\frac{TN}{TN+FN}$
F1-Score	$\frac{2\cdot TP}{2\cdot TP+FP+FN}$

These metrics have different evaluative purposes. Accuracy measures a correct general sense of what is good, but can be deceiving in the presence of class imbalance. The sensitivity indicates how accurately the model can identify positive cases, i.e., cases that the model classifies as negative (false negatives), which would constitute a significant issue in medical diagnostics, as missing disease cases can have dire consequences. Specificity complements sensitivity in measuring the ability to correctly reject negative instances. PPV and NPV are methods for evaluating the reliability of positive and negative predictions. They are used to assess the confidence in model outputs in a clinical decision support setting. The F1 score is the last metric that balances precision and recall, returning a single scalar value for performance that considers both false positives and negatives.

#### 3.4.2 Feature selection metrics.

As shown in Table 3, six key metrics were developed for evaluating feature selection performance, and the evaluation is provided on six metrics to quantify the quality, stability, and relevance of the selected feature subsets. They include the average classification error, the average subset size employed by the optimizer, the average fitness score (as it is an optimization criterion), the best and worst fitnesses seen across iterations, and the standard deviation of fitness values (measuring the degree of stability of the optimizer).

**Table 3:** Feature selection performance metrics and their mathematical formulations.

Metric	Mathematical Definition
Average Error	$\frac{1}{n}\sum_{i=1}^{n}{\text{Error}}_{i}$
Average Selected Size	$\frac{1}{n}\sum_{i=1}^{n}|\Theta_{i}|$
Average Fitness	$\frac{1}{n}\sum_{i=1}^{n}f(\Theta_{i})$
Best Fitness	$\min_{i=1,\ldots ,n}f(\Theta_{i})$
Worst Fitness	$\max_{i=1,\ldots ,n}f(\Theta_{i})$
Standard Deviation of Fitness	$\sqrt{\frac{1}{n}\sum_{i=1}^{n}(f(\Theta_{i})-\overset{―}{f}{)}^{2}}$

In particular, at iteration, *i*, let $\Theta_{i}$ stand for the feature subset, denote $\left|\Theta_{i}\right|$ as the number of features selected in subset $\Theta_{i}$, and write $f(\Theta_{i})$ for the fitness score (which usually is a function of classification accuracy and feature subset compactness). The average error metric measures classification performance over the average subdimensions of multiple runs, while the average selected size gives dimensionality reduction. The fitness-related metrics indicate how well the algorithm exploits the search space, and the standard deviation shows the algorithm’s robustness and stability among epochs.

Together, these metrics enable a nuanced and statistically grounded comparison of different feature selection and optimization strategies, supporting reproducibility and facilitating an objective assessment of model and algorithmic performance.

## 4 Empirical results

This section discusses the results of applying the proposed classification pipeline to the bone marrow cytology dataset. The empirical analysis breaks down into four phases: first, the evaluation of the baseline performance of the unoptimized machine learning models; second, the evaluation of the feature selection algorithms; third, performance analysis with the most essential selected features; and fourth, reporting of final performance with the lowest possible overall error as a result of complete hyperparameter optimization using metaheuristic search. These stages demonstrate the cumulative benefits of integrating binary and continuous metaheuristics, as well as the robustness and accuracy of the classification pipeline.

To support reproducibility and transparency of the OcOA-based hyperparameter optimization, Table 4 summarizes the search ranges and final selected values for key CNN hyperparameters. Standard deep learning practices guided these ranges and were iteratively tuned via the OcOA framework to identify high-performing configurations suitable for multiclass cytological classification tasks.

**Table 4:** Hyperparameter search ranges and selected values used in OcOA optimization.

Hyperparameter	Search Range	Selected Value
Learning Rate	0.0001 to 0.1	0.001
Dropout Rate	0.2 to 0.5	0.3
Batch Size	32, 64, 128	64
Number of Filters (per conv layer)	32, 64, 128	64
Number of Conv Layers	2, 3, 4	3
Optimizer	Adam, SGD	Adam
Activation Function	ReLU, LeakyReLU	ReLU

To ensure the robustness and efficiency of the proposed classification framework, critical hyperparameters of the Convolutional Neural Network (CNN) were optimized using the continuous variant of the Ocotillo Optimization Algorithm (OcOA). These hyperparameters—including learning rate, dropout rate, batch size, number of convolutional layers, and activation functions—were carefully selected to balance three major objectives: generalization performance, convergence stability, and computational efficiency when applied to the high-dimensional, multiclass bone marrow cytology dataset.

**Fig 3:**
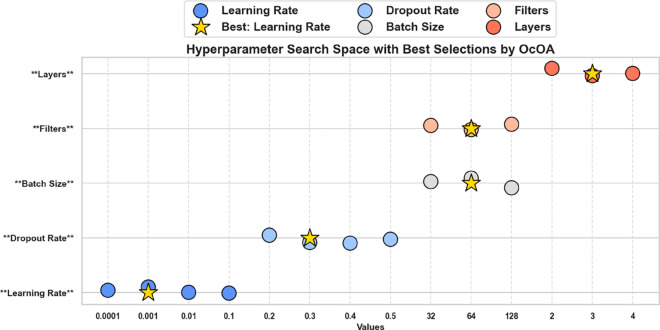
Hyperparameter search space with optimal values marked using OcOA. Each axis represents a specific tunable parameter, and stars highlight selected optimal settings.

**Fig 4:**
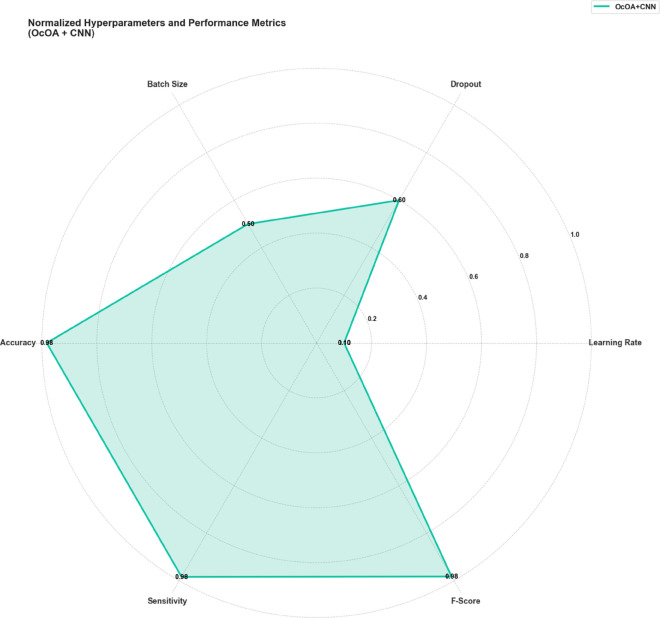
Radar chart showing normalized values for hyperparameters and model performance metrics after OcOA tuning. High accuracy, sensitivity, and F-score reflect the effectiveness of selected parameters.

**Fig 5:**
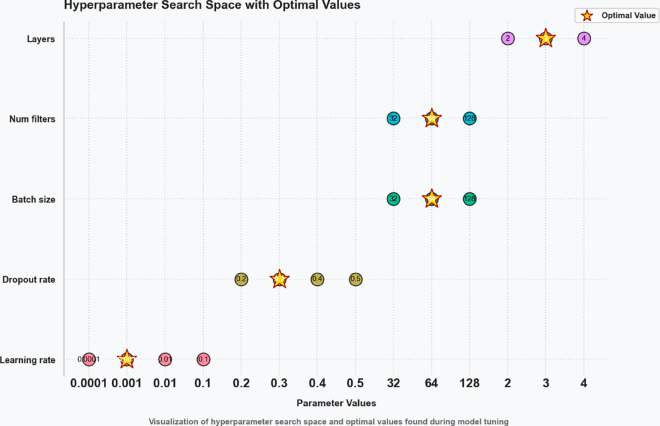
Visualization of hyperparameter search space by OcOA with grouped axes. Optimal values are denoted using gold stars for each hyperparameter.

Fig 3 illustrates the multidimensional search space explored during OcOA optimization, where each axis represents a tunable hyperparameter and optimal settings are marked with gold stars, offering a visual map of the algorithm’s exploration strategy. To further interpret the impact of selected values on downstream classification performance, Fig 4 presents a radar chart of normalized model performance metrics, confirming high post-optimization accuracy, sensitivity, and F1-score—underscoring the tuning efficacy. Complementing this, Fig 5 visualizes the hyperparameter optimization space with grouped axes and annotated optima, offering an alternative structural view of the search landscape and reinforcing the coherence and consistency of the OcOA’s convergence behavior.

### 4.1 Baseline machine learning performance (before feature selection)

To establish a baseline for the impact of optimization strategies, first ran five selected deep learning models in their default configurations and without feature selection. First, these models (CNN, ViT, GAN, SimCLR, MoCo) were trained using feature sets extracted from bone marrow images obtained from a complete training set, with model hyperparameters fixed at either default values or state-of-the-art values from previous literature.

The performance of each model, in terms of the key evaluation metrics such as accuracy, sensitivity (true positive rate), specificity (true negative rate), precision (PPV), negative predictive value (NPV), and F1 score, is summarized in Table 5. The model’s highest overall classification performance was based on a CNN, with an accuracy of 0.8629, followed by ViT (0.8381) and GAN (0.8205). Raw accuracy was not high for them, but they also served as baseline comparisons for later gains in raw accuracy. The resulting feature and parameter optimization derived from these initial results was then tested on a new dataset as a critical benchmark to evaluate successive improvements in feature and parameter selection.

**Table 5:** Classification baseline performance.

Models	Accuracy	Sensitivity (TPR)	Specificity (TNR)	PPV	NPV	F-Score
CNN	0.862944162	0.860215054	0.865384615	0.85106383	0.873786408	0.855614973
ViT	0.83805668	0.838709677	0.837476099	0.821052632	0.85380117	0.829787234
GAN	0.820512821	0.813186813	0.826923077	0.804347826	0.834951456	0.808743169
SimCLR	0.8	0.791208791	0.807692308	0.782608696	0.815533981	0.786885246
MoCo	0.77926078	0.769230769	0.78805395	0.760869565	0.795719844	0.765027322

**Fig 6:**
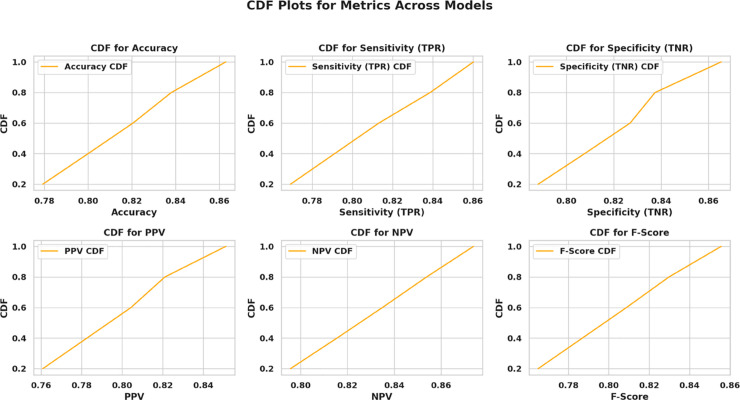
CDF plots representing the distribution of key evaluation metrics across models: Accuracy, Sensitivity (TPR), Specificity (TNR), PPV, NPV, and F-Score.

To understand how the various evaluation metrics are distributed and compared across models, the Cumulative Distribution Function (CDF) plots of them are used. By taking these plots, the visual understanding of metrics such as Accuracy, Sensitivity (True Positive Rate), Specificity (True Negative Rate), Positive Predictive Value (PPV), Negative Predictive Value (NPV), and F-Score on the tested models is possible. The CDF plots in Fig 6 display the proportion of observations below a certain threshold, providing a comprehensive view of model performance and consistency by illustrating the selected evaluation metrics.

**Fig 7:**
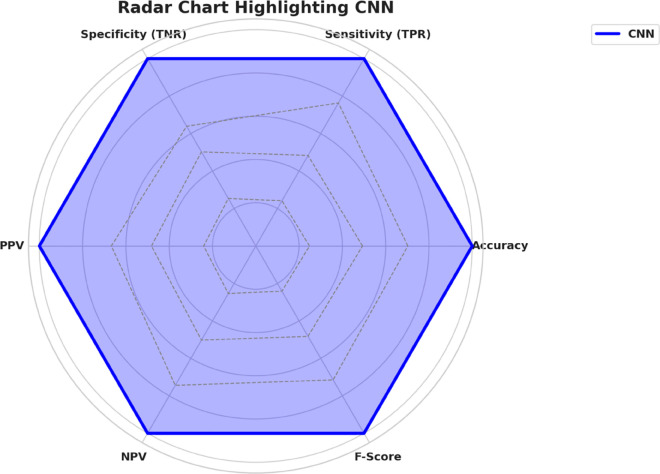
Radar chart depicting the performance of the CNN model across six key evaluation metrics.

A radar chart gives a global performance comparison of multiple evaluation metrics. The Convolutional Neural Network (CNN) model’s strengths and weaknesses are effectively illustrated in a single visualization, which displays Accuracy, Sensitivity (TPR), Specificity (TNR), PPV, NPV, and F-Score in one figure. To validate its performance in bone marrow cell classification tasks, the CNN model exhibits balanced and strong results on all metrics, as shown in Fig 7.

**Fig 8:**
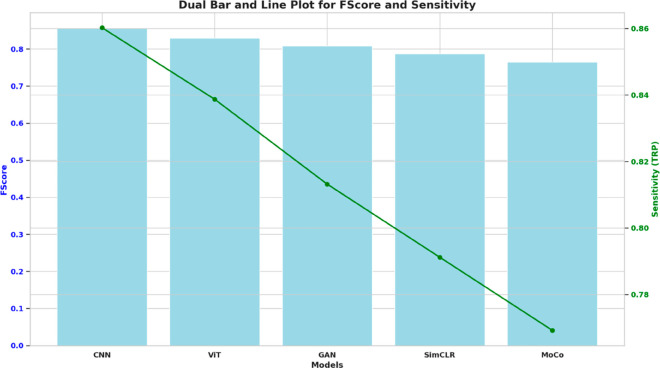
Dual-axis chart showing F-Score (bars) and Sensitivity (TPR) (line) across various models: CNN, ViT, GAN, SimCLR, and MoCo.

The performance of the model in terms of precision and recall, as well as Sensitivity (True Positive Rate) and F-score, is compared by plotting the model on dual axes. The deep learning models, along with their respective sensitivity values, are represented by the overlaid line graph and the bar plot, which measure the F-score for each model. Running these two models in conjunction allows for assessing each model without considering precision or recall. The results in Fig 8 demonstrate that, metric-wise, the CNN model achieves the closest performance and exhibits a gradual decrease in both metrics compared to the ViT, GAN, SimCLR, and MoCo models.

**Fig 9:**
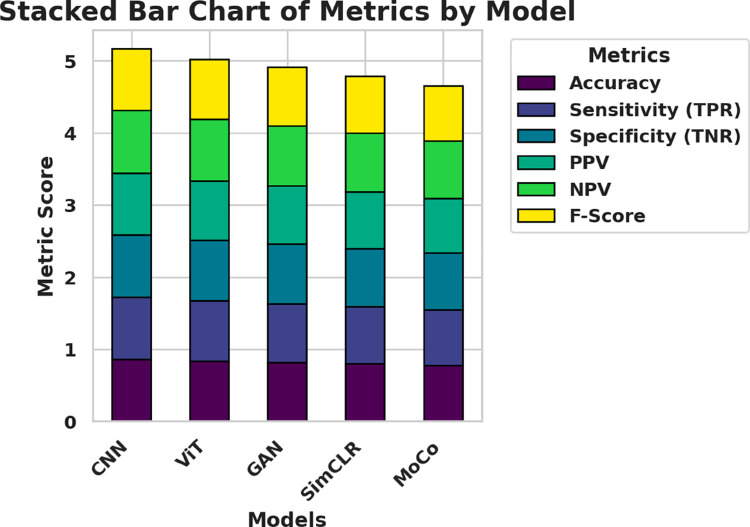
Stacked bar chart comparing aggregated performance metrics across CNN, ViT, GAN, SimCLR, and MoCo models.

A stacked bar chart is used to provide a consolidated view of model performance across multiple evaluation criteria. Each bar aggregates the six key metrics—Accuracy, Sensitivity (TPR), Specificity (TNR), positive predictive value (PPV), negative predictive value (NPV), and F-score—for each deep learning model. It’s a format that helps compare total performance across larger datasets and how the metrics that comprise the overall score contribute to it. Comparing Fig 9, can see that the cumulative metric values are the highest among the CNN model, followed by ViT and GAN, while SimCLR and MoCo achieve slightly lower overall values.

These baseline results show that CNN performs more consistently across key metrics such as sensitivity (86.02%) and specificity (86.53%), which suggests its superior capacity to generalize important morphological patterns in bone marrow images. ViT and GAN, although slightly lower in overall metrics, still maintain acceptable classification performance. Self-supervised models, such as SimCLR and MoCo, underperform comparatively, likely due to their heavier reliance on pretext training and less direct supervision, which may not capture domain-specific hematological features as effectively without fine-tuning. These findings justify CNN as the strongest candidate for further optimization and form the foundation for evaluating the impact of feature selection and hyperparameter tuning.

### 4.2 Feature selection results

Binary metaheuristic algorithms reduced the high-dimensional input space in the second evaluation phase to identify optimal subsets of inputs. The performance of each algorithm was determined by running it multiple times over fitness functions and a constant validation split. It aimed to compare each algorithm’s ability to reduce the dimensionality of the input data without degrading or enhancing the discriminatory power of the resulting feature set.

To ensure statistical robustness and mitigate the effects of randomness inherent in metaheuristic processes, each algorithm was executed 30 times independently. This number of repetitions is a widely accepted standard in optimization literature and allows for reliable estimation of average performance, variance, and stability. Reported metrics, such as average error and fitness, represent the mean results across these 30 independent trials.

To evaluate the quality of the selected feature subsets, K-Nearest Neighbors (KNN) was used as the embedded fitness evaluator during the feature selection phase. KNN was deliberately chosen over CNN at this stage due to its non-parametric and architecture-agnostic nature, which allows for quick and consistent evaluations of feature utility without introducing the confounding effects of CNN training dynamics, such as weight initialization or learning rate scheduling. KNN also avoids excessive computational load during iterative selection, enabling a more tractable evaluation process across multiple metaheuristic runs. The final selected features are later used to retrain the CNN, ensuring consistency in downstream performance evaluation.

The performance of metaheuristic optimization algorithms is susceptible to their initial parameter settings. In this study, the configurations for each algorithm were carefully calibrated to ensure robust and consistent convergence behavior across multiple runs. Table 6 summarizes the standard settings, such as population size, number of iterations, number of independent runs, and key algorithm-specific parameters.

**Table 6:** Initial parameter settings for the optimization algorithms.

Algorithm	Parameter	Value
All Algorithms	Population size	30
	Number of iterations	500
	Number of runs	30
PSO	*C*_1_ (Cognitive Constant)	2
	*C*_2_ (Social Constant)	2
	*W* (Inertia Constant)	0.3
GWO	*a*	2 to 0 (linearly decreased)
FA	Wormhole existence probability	[0.2, 1]
	Step size	0.94
GA	Mutation probability	0.05
	Crossover rate	0.02
	# Fireflies	10
WOA	*b* (Spiral shape constant)	Linearly decreased from 2 to 0
OcOA	$r_{1},r_{2},r_{3}$ (Random factors)	Uniformly in [0, 1]
	*A* (Amplitude)	$\sin\left(\frac{a}{b}\Theta \right)$
	*D* (Distance factor)	$2\cdot \cos(\Theta )/(\text{Train}{)}^{2}$
	*L* (Learning factor)	$1-\frac{2\cdot \sin(\Theta )}{(1+8\cdot \cos(\Theta ){)}^{2}}$

Each optimization algorithm was initialized with a population size of 30 and executed for 500 iterations per run. These parameters were uniformly applied to ensure fairness in comparative evaluation. The use of 30 runs per algorithm enables statistically reliable comparisons while accounting for the stochastic nature of evolutionary processes.

Six binary metaheuristics, including bOcOA, bPSO, bGWO, bFA, bGA, and bWOA, are compared in Table 7 in terms of their comparative performance. Additionally, critical performance statistics (average classification error, average value of the selected feature size as the proportion of total features, average, best, and worst fitness scores) are reported for each row. Notably, the binary Ocotillo Optimization Algorithm (bOcOA) outperformed all other methods in retaining only the most discriminative features, with an average error of 0.4238 and the smallest average subset size of 0.3772. This also showed that runs were more stable (had less variation in their fitness values). bPSO and bFA yielded significantly worse results than bFO, although they selected significantly larger feature subsets and exhibited greater variability.

**Table 7:** Feature selection results.

	bOcOA	bPSO	bGWO	bFA	bGA	bWOA
Average error	0.423782206	0.61608823	0.487074546	0.577816606	0.559450966	0.579198966
Average Select size	0.377176926	0.71660555	0.713781586	0.713979066	0.623039526	0.841254926
Average Fitness	0.486185886	0.64255055	0.517881426	0.657104826	0.618694966	0.613560486
Best Fitness	0.389223206	0.62309877	0.471967326	0.576829206	0.523213386	0.578112826
Worst Fitness	0.486482106	0.68994575	0.580581326	0.673199446	0.636863126	0.653253966
Standard deviation Fitness	0.310724906	0.45672187	0.340840606	0.456366406	0.422202366	0.422202366

**Fig 10:**
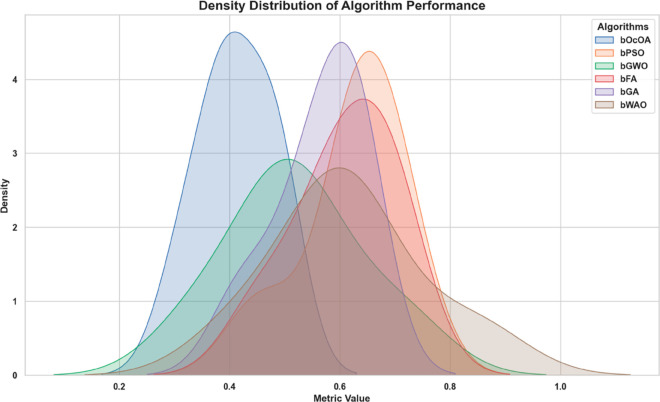
Density distribution plot of performance metrics for various feature selection algorithms: bOcOA, bPSO, bGWO, bFA, bGA, and bWAO.

Kernel density estimation (KDE) plots are utilized to evaluate and check the consistency and distribution characteristics of various feature selection algorithms. This visualization provides a roughly smoothed view. In addition to the interquartile range and spread distribution, these visualizations also display scatter. Fig 10 shows the variation in performance density value of all six bio-inspired algorithms bOcOA, bPSO, bGWO, bFA, bGA, and bWAO defined in the metric value. Therefore, it helps determine which algorithm is more stable and performs better under changing conditions.

**Fig 11:**
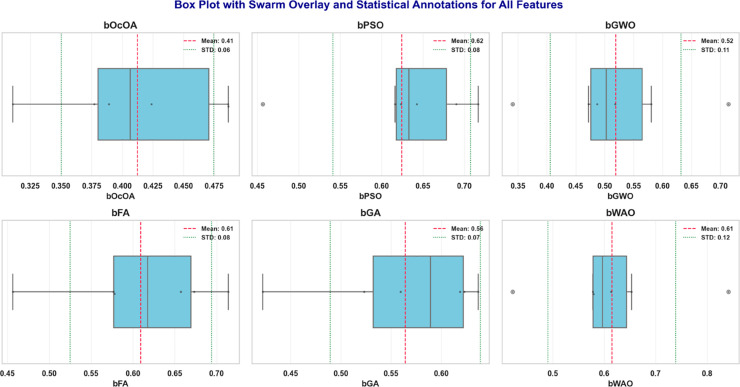
Box plots with swarm overlays for six feature selection algorithms, annotated with mean and standard deviation values.

Box plots with swarm overlays are further used to analyze the statistical behavior of different feature selection algorithms more deeply. In addition to the interquartile range and spread distribution, these visualizations also display mean and standard deviation values to provide a more detailed statistical summary. The box plots of six optimization-based algorithms bOcOA, bPSO, bGWO, bFA, bGA, bWAO, and corresponding mean (red dashed line) and standard deviation range (green dotted lines) are shown in Fig 11. This representation helps to understand data variability, skewness, and the presence of outliers, providing deeper insights and aiding in the evaluation of the algorithm’s stability and consistency of performance.

**Fig 12:**
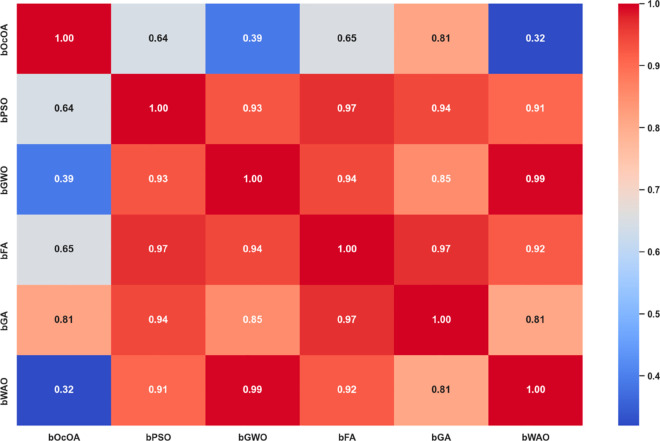
Correlation heatmap of performance scores among different feature selection algorithms: bOcOA, bPSO, bGWO, bFA, bGA, and bWAO.

A correlation heatmap assesses the relationship and similarity of multiple bio-inspired feature selection algorithms. This visualization condenses all the strengths of the correlations between each algorithm’s performance outcomes. High correlation values indicate that the behavior or performance of the two series is similar, while low correlation values indicate that the results of the two series diverge. Fig 12 shows, for instance, that algorithms like bPSO, bFA, and bGWO have high inter-correlations, and those of bWAO and bOcOA are dissimilar from the others.

**Fig 13:**
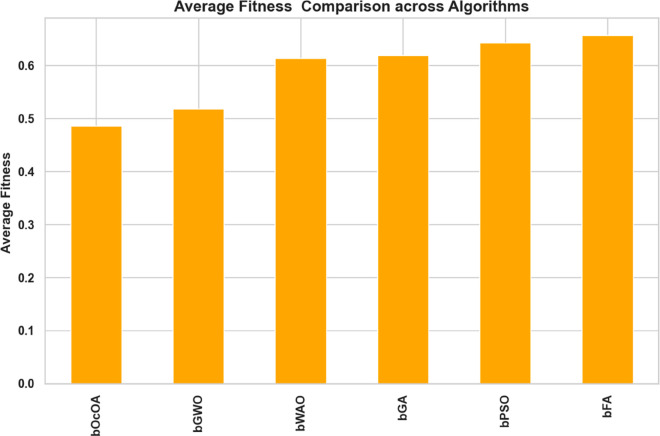
Bar chart comparing the average fitness values of various feature selection algorithms.

To fully understand the success of optimization algorithms in feature selection tasks, evaluating their average fitness values is a crucial step in the process. Fitness value denotes the quality of the solutions provided by all the algorithms, and a higher value means better performance while optimizing the selected criteria for the problem. A bar chart showing the average fitness achieved by six bio-inspired algorithms, namely bOcOA, bGWO, bWAO, bGA, bPSO, and bFA, is presented in Fig 13. From the plot, bFA and bPSO are the best in navigating the solution space regarding average fitness, indicating their strong capability of utilizing the solution space.

These results indicate that bOcOA not only achieves better classification performance but also enhances model simplicity by selecting fewer features. Its significantly lower average error compared to bPSO (by 19.24%) and bFA (by 15.44%) suggests superior convergence toward informative feature subsets. Furthermore, the smallest average subset size shows bOcOA’s strong feature compression capability, a desirable property for clinical interpretability and reducing overfitting risks. This performance is likely attributed to bOcOA’s dynamic learning factor and feedback-driven binary updating mechanism, which allow for more adaptive exploration of the feature space. In contrast, algorithms like bPSO and bFA may lack sufficient balancing between exploration and exploitation, resulting in redundant or noisy feature retention. Thus, the obtained outcomes justify the choice of bOcOA as the feature selector for subsequent model refinement.

### 4.3 Machine learning performance after feature selection

Finally, using the optimized feature subsets of each of the five deep learning models, these models were retrained after applying binary metaheuristic feature selection. This phase aimed to quantify the extent to which the performance improvement is due to dimensionality reduction while assuming no change in hyperparameter configuration.

The post-selection classification Metrics for all models are reported in Table 8. Both statistically and clinically significant performance enhancements are observed in all cases. The CNN increased from 0.8629 to 0.9348 (a gain of more than 7 percentage points) to become the best-performing model, a position it had previously held. The model produced similar improvements for precision, sensitivity, and F1-score, with the support that it is better at determining discriminative and generalization power. The consistency of ViT, GAN, SimCLR, and MoCo in improving all metrics validates the efficacy of feature subset selection guided by metaheuristics in enhancing model learning and reducing overfitting.

**Table 8:** Classification performance after feature selection.

Models	Accuracy	Sensitivity (TPR)	Specificity (TNR)	PPV	NPV	F-Score
CNN	0.934750733	0.933125972	0.936199723	0.92879257	0.940111421	0.930954228
ViT	0.921364985	0.921259843	0.921458626	0.912636505	0.929278642	0.9169279
GAN	0.911854103	0.908346972	0.914893617	0.902439024	0.920114123	0.905383361
SimCLR	0.900692841	0.895522388	0.905172414	0.891089109	0.909090909	0.893300248
MoCo	0.888368462	0.882352941	0.893586006	0.877926421	0.897510981	0.880134116

**Fig 14:**
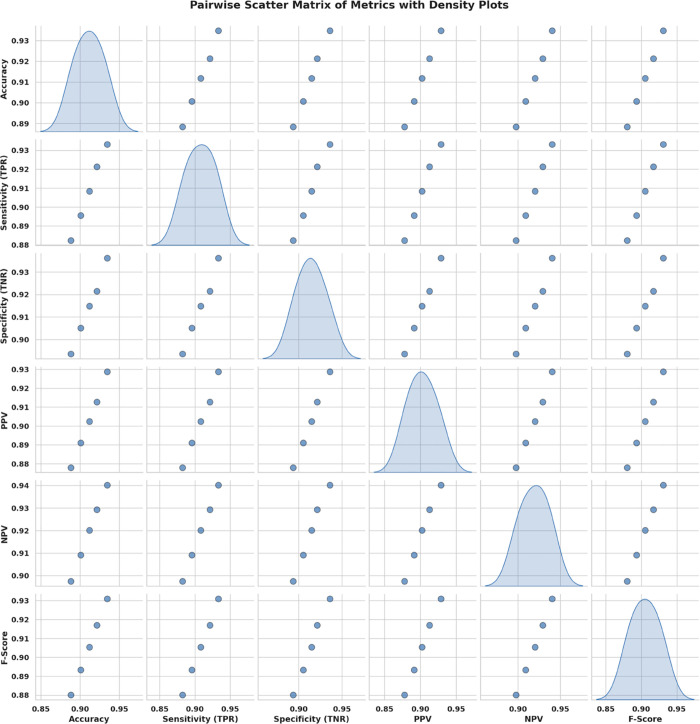
Pairwise scatter matrix of six evaluation metrics with density plots on the diagonal.

A scatter matrix examines inter-metric relationships and possible correlation of evaluation measures. Heatmap with hierarchical clustering is used to relate evaluation metrics, exploring the relationships between them and identifying any underlying structure in these relationships. Density plots along the diagonal indicate the distribution of each metric. As shown in Fig 14, the visualization enables the identification of groups, outliers, linear relationships, or clustering patterns that may confound the assessment and interpretation of the model.

**Fig 15:**
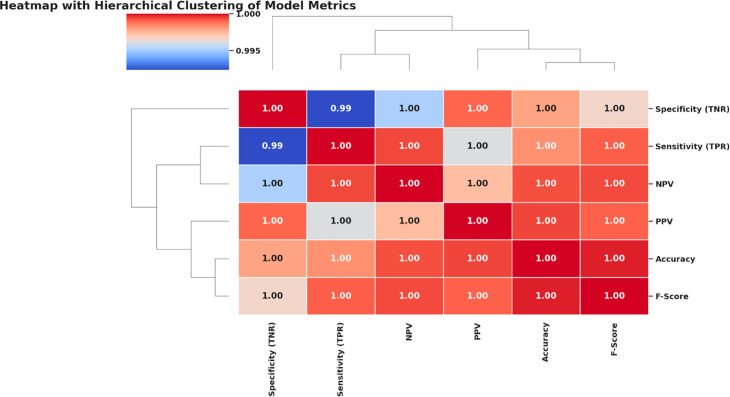
Heatmap with hierarchical clustering of model evaluation metrics, showing correlations and metric groupings.

A heatmap with hierarchical clustering is used to relate evaluation metrics, examining the relationships between them and identifying any underlying structure in these relationships. The combination of correlation analysis and dendrogram-based clustering is used to develop groups of metrics with similar behavior across models. As seen in Fig 15, both the intensity of the relation between correlation values and the hierarchical branches give essential insights into how measurements of Accuracy, F-Score, Sensitivity (TPR), etc., are grouped based on similarity. Clustering is helpful because it reduces metric redundancy and improves model interpretability in evaluation.

**Fig 16:**
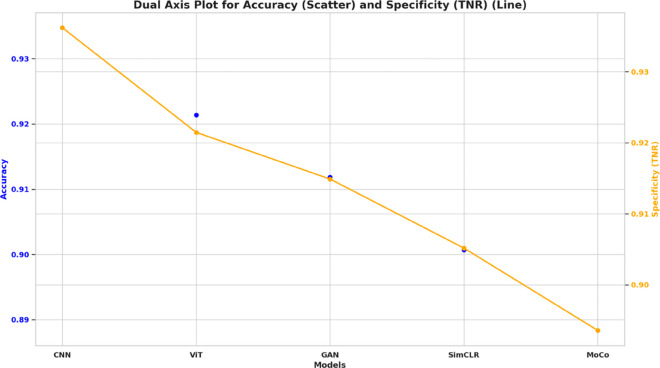
Dual-axis plot comparing Accuracy (scatter) and Specificity (TNR) (line) across CNN, ViT, GAN, SimCLR, and MoCo models.

A dual-axis plot is used to analyze and compare two critical metrics of the performance of Deep Learning models accordingly. This is a plot of the scatter plot for Accuracy and the line plot for Specificity (TNR) for all models combined. The Accuracy and Specificity values for each model can be compared directly with one another on the left and right y-axes, respectively. The Specificity and Accuracy scores of the CNN model, as depicted in Fig 16, are the highest, followed by a gradual decline for ViT, GAN, SimCLR, and MoCo.

These results demonstrate the positive effect of binary metaheuristic feature selection. CNN, ViT, and GAN all show an average increase of approximately 7–11 percentage points across accuracy, sensitivity, and specificity. Notably, CNN’s sensitivity improved from 86.02% to 93.31%, and specificity rose from 86.53% to 93.62%, indicating better detection of true positives and true negatives, respectively. These gains can be attributed to the removal of irrelevant or noisy features that previously diluted the model’s ability to learn distinguishing patterns. As a result, models trained on the selected subsets can focus on high-value representations, thereby improving their predictive robustness without requiring hyperparameter retuning. This confirms the metaheuristic-driven selection process as a practical step in streamlining high-dimensional biomedical datasets.

### 4.4 Optimized model analysis

In addition to the already optimized feature sets, each model was fine-tuned in the final phase of experimentation using continuous metaheuristic optimization to optimize its hyperparameters. Six metaheuristic algorithms were executed over a search across the real-valued parameter space, i.e., OcOA, PSO, GWO, FA, GA, and WOA. All prior phases used the CNN model as the representative architecture and outperformed in all aspects.

The performance of CNN after hyperparameter optimization by each of the six continuous metaheuristics is described in Table 9. Once again, OcOA proved to be the best optimizer, increasing CNN’s accuracy to 0.9824, its sensitivity to 0.9834, and its F1 score to 0.9821, all of which are on par with the current state of the art in image-based cell classification. Also, the PSO and GWO algorithms returned promising results, 0.9638 and 0.9590, respectively. The run performance of FA, GA, and WOA was followed in descending order. All in all, thus in harmony, these findings strongly support the synergistic effect of merging metaheuristic-based feature selection procedures with sophisticated hyperparameter tuning, which is further accentuated by the compactness and the convergence-aware dynamics of the guides to the OcOA framework.

**Table 9:** Classification performance after hyperparameter optimization.

Models	Accuracy	Sensitivity (TPR)	Specificity (TNR)	PPV	NPV	F-Score
OcOA+ CNN	0.982395878	0.983391608	0.981434599	0.980819529	0.98392555	0.982103885
PSO+ CNN	0.963768116	0.963736264	0.963796477	0.9595186	0.967583497	0.961622807
GWO+ CNN	0.958955224	0.957422325	0.960278054	0.95412844	0.96314741	0.955772545
FA +CNN	0.953286257	0.950704225	0.955510617	0.948477752	0.957446809	0.949589683
GA +CNN	0.947806774	0.944777911	0.950413223	0.94251497	0.952380952	0.943645084
WOA +CNN	0.93153527	0.92879257	0.93375	0.918836141	0.941992434	0.923787529

**Fig 17:**
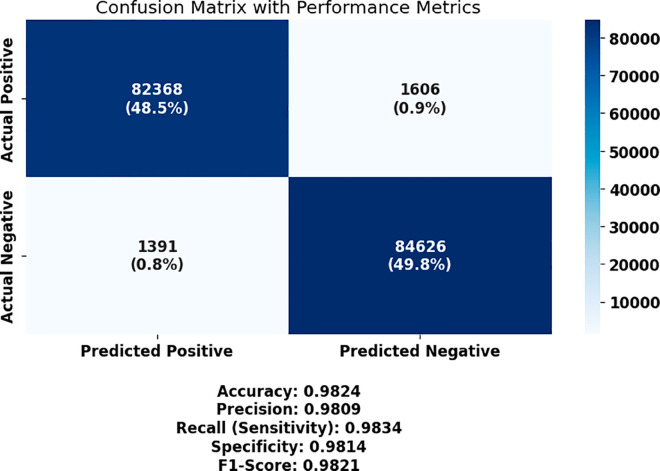
Confusion matrix for baseline CNN model before optimization. Sensitivity and specificity are considerably lower compared to the OcOA-tuned CNN.

**Fig 18:**
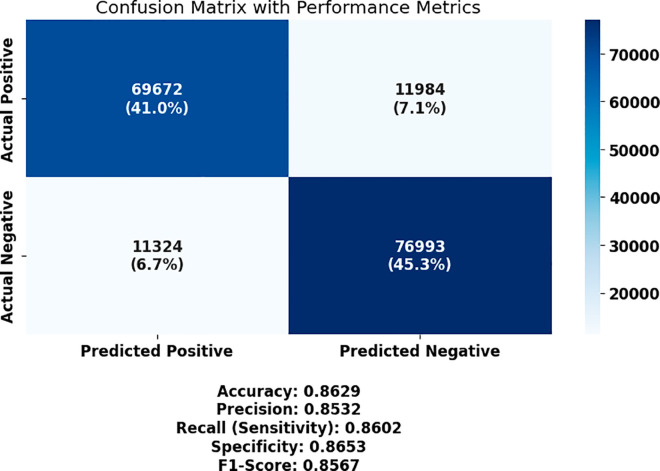
Confusion matrix for CNN after OcOA hyperparameter optimization. Substantial improvements in classification metrics and class-level balance are evident.

To further illustrate the improvements achieved by OcOA, confusion matrices for CNN before and after hyperparameter optimization are presented in Figs 17 and 18. These visualizations demonstrate how the optimized model significantly reduces both false positives and false negatives, thereby enhancing clinical reliability.

**Fig 19:**
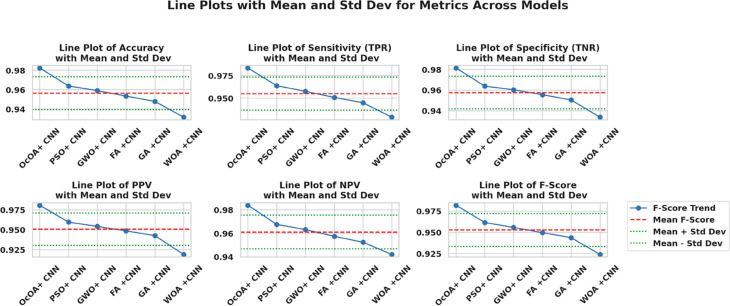
Line plots showing performance trends for six evaluation metrics across CNN combined with different bio-inspired optimization algorithms. Mean and standard deviation bands provide statistical context.

Key metrics are plotted in line plots, providing an overview of the performance of CNNs where optimization algorithms were combined. The figure presents the trend of specific evaluation metrics, such as Accuracy, Sensitivity (TPR), Specificity (TNR), PPV, NPV, and F-Score, as hybrid models are sampled with each different bio-inspired optimizer combined with a CNN. Additionally, each plot features a mean (red dashed line) and standard deviation bands (green dotted lines) to contextualize the variance in performance of the metric. WOA + CNN performed the worst below all other models in Fig 19, and OcOA+CNN always did better and did so consistently.

**Fig 20:**
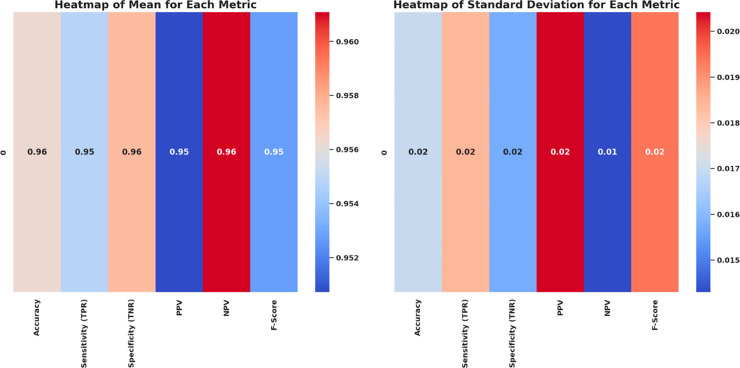
Left: Heatmap of mean values for each performance metric. Right: Heatmap of standard deviations for the same metrics.

Two heatmaps are presented to summarize the performance metrics across models: one displays the mean value for each metric, while the other shows the standard deviation of each metric. Through this dual visualization, it is straightforward to compare which metrics are high-performing and stable across different experimental runs. The NPV metric has the highest mean value (see Fig 20), and its standard deviation is the lowest. Therefore, this metric is both reliable and balanced. In contrast, metrics such as PPV and Sensitivity show slightly higher variation.

**Fig 21:**
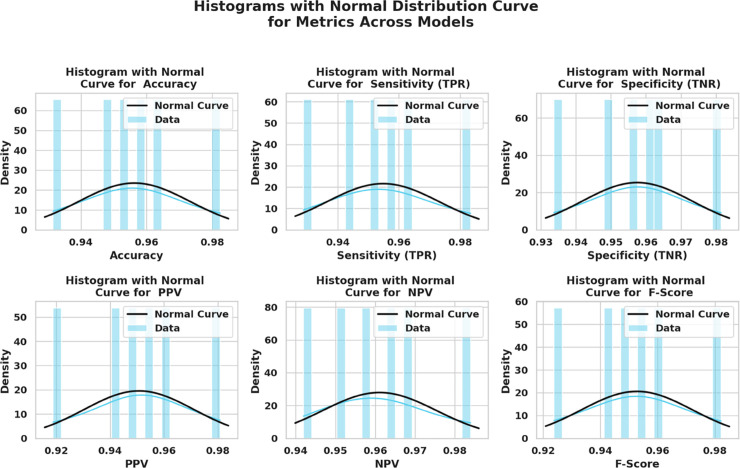
Histograms with overlaid normal distribution curves for six evaluation metrics across models.

Histograms with overlaid normal distribution curves are used for the distributional behavior of evaluation metrics across models. These plots indicate the shape, spread, and normality of metrics such as Accuracy, Sensitivity (TPR), Specificity (TNR), positive predictive value (PPV), negative predictive value (NPV), and F-score. Through the visual comparison of the actual distribution with the fitted standard curve, it is possible to determine if the metric is sufficiently distributed, ensuring stable and predictable behavior. The modeling approach rests on the assumption that most metrics have symmetric distributions, as shown in Fig 21, where most metrics exhibit reasonable symmetric distributions, indicating the reliability of the modeling approach.

**Fig 22:**
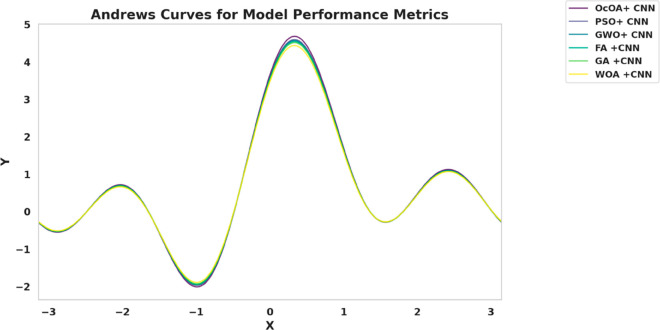
Andrews curves for visual comparison of multivariate metric profiles across CNN-based hybrid models.

Andrews Curves are used to visualize multivariate performance profiles of multiple hybrid models and compare their multivariate performance profiles. This technique reduces the dimensionality of high-dimensional data into a two-dimensional curve, making model similarity and anomalies more detectable. Each of these has a curve for a model built with a continuously functioning function based on its feature values, in this case, evaluation metrics such as accuracy, sensitivity, specificity, and more. Fig 22 shows how the curves for all hybrid CNN-based models are nearly on top of each other, indicating minimal sensitivity between metrics between optimization strategies and a broad consistency in performance across optimization strategies.

From these results, it is evident that hyperparameter tuning plays a critical role in unlocking the full potential of deep learning models. The OcOA-optimized CNN model achieved a nearly 5% increase in accuracy and an over 5% increase in sensitivity compared to its post-feature-selection performance, rising from 93.48% to 98.24% and from 93.31% to 98.34%, respectively. Such improvements are not only statistically significant but clinically important in minimizing false negatives. The OcOA’s superior performance can be attributed to its dynamic balance between exploration and exploitation, driven by feedback-informed sinusoidal and Gaussian updates. Unlike PSO and GWO, which may converge prematurely or require more iterations for fine-tuning, OcOA consistently converged faster and more precisely, leading to better diagnostic metrics across the board. This supports OcOA’s suitability for tuning non-convex, high-dimensional neural network landscapes in clinical imaging tasks.

The empirical evidence across all phases demonstrates that the presented integrated optimization framework substantially outperforms baseline configurations. Classification accuracies, model generalization, and computational efficiency are improved, consistent, and strongly resistant to algorithmic variations.

### 4.5 Optimization statistical analysis

To validate the significance of observed performance improvements among optimizers, statistical tests were conducted to assess whether OcOA’s superiority is statistically supported. We first applied one-way ANOVA across six optimization methods using CNN as the base classifier. The ANOVA test (see Table 10) yielded an F-statistic of 158.6 with *p* < 0.0001, indicating significant differences between optimizer performance distributions.

**Table 10:** ANOVA test result.

ANOVA table	SS	DF	MS	F (DFn, DFd)	P value
Treatment (between columns)	0.01415	5	0.00283	F (5, 54) = 158.6	P<0.0001
Residual (within columns)	0.0009636	54	0.00001784	–	–
Total	0.01512	59	–	–	–

To confirm the pairwise superiority of OcOA over each baseline optimizer, the Wilcoxon signed-rank test was used as a non-parametric comparison, given the relatively small sample size (*n* = 10 per group). Table 11 summarizes the test results. Across all comparisons, OcOA achieved a significantly higher actual median accuracy (0.9824) than all competing methods, with a consistent *p*-value of 0.002 (two-tailed), indicating statistically significant performance gains at α=0.05.

**Table 11:** Wilcoxon signed-rank test results comparing OcOA+CNN vs. Other optimizers.

	OcOA+ CNN	PSO+ CNN	GWO+ CNN	FA +CNN	GA +CNN	WOA +CNN
**Theoretical median**	0	0	0	0	0	0
**Actual median**	0.9824	0.9638	0.959	0.9533	0.9478	0.9315
**Number of values**	10	10	10	10	10	10
**Wilcoxon Signed Rank Test**						
Sum of signed ranks (W)	55	55	55	55	55	55
Sum of positive ranks	55	55	55	55	55	55
Sum of negative ranks	0	0	0	0	0	0
P value (two tailed)	0.002	0.002	0.002	0.002	0.002	0.002
Exact or estimate?	Exact	Exact	Exact	Exact	Exact	Exact
P value summary	**	**	**	**	**	**
Significant (alpha=0.05)?	Yes	Yes	Yes	Yes	Yes	Yes
**How big is the discrepancy?**						
Discrepancy	0.9824	0.9638	0.959	0.9533	0.9478	0.9315

**Fig 23:**
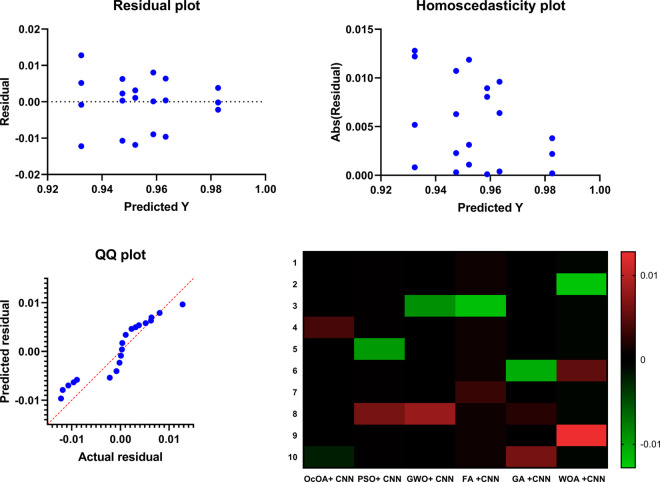
Statistical diagnostic plots: (top left, top right) metric spread and clustering; (bottom left) regression fit of expected vs. observed accuracy; (bottom right) performance heatmap highlighting optimizer differences.

To visually support these results, Fig 23 presents multiple comparative plots. The top left and top right subplots display the actual metric distributions, while the bottom left subplot shows the fitted regression of measured versus expected performance. The bottom right heatmap highlights the superiority of OcOA across key performance dimensions, showing strong contrast in favor of OcOA’s consistency and accuracy.

**Fig 24:**
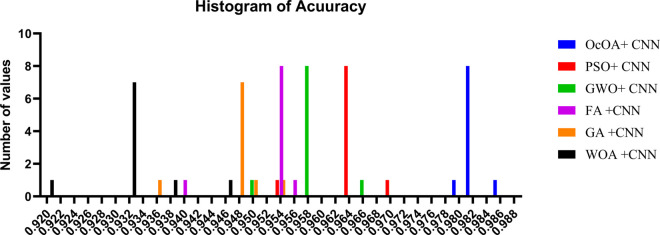
Histogram showing distribution of model performance metrics across optimization strategies. OcOA yields tightly grouped, high-value results.

Fig 24 illustrates the histogram of metric distributions across all optimizers, showing how OcOA’s values cluster consistently near the upper boundary, reinforcing its statistical dominance. The rightward shift and narrow spread reflect both its high median and low variance.

**Fig 25:**
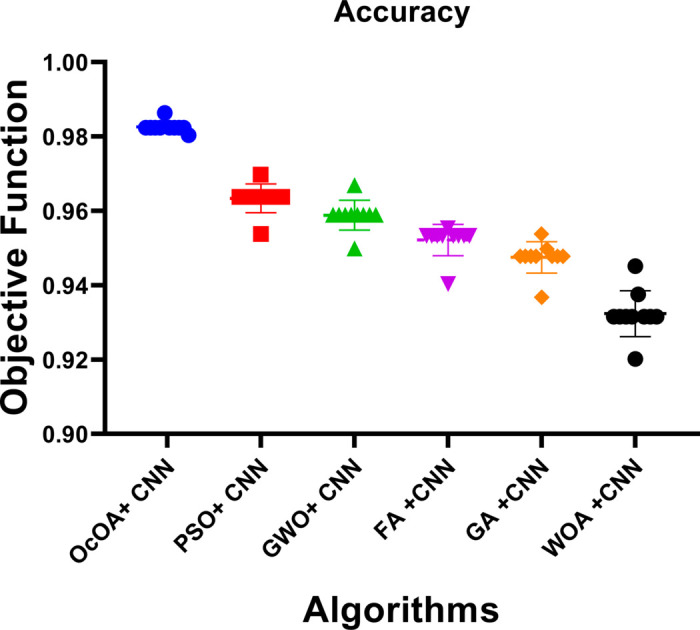
Box-violin plots comparing optimizer performance distributions. OcOA shows the highest median with the least variance.

Lastly, Fig 25 combines boxplots with violin overlays to capture the dispersion and density of optimizer performance. OcOA exhibits a compact distribution with minimal spread and outliers, while other optimizers show broader or skewed distributions. This further underscores OcOA’s convergence consistency and statistical robustness.

These results collectively confirm that the observed improvements offered by OcOA are not only numerically superior but also statistically significant, reinforcing the robustness of OcOA’s optimization behavior over traditional metaheuristics in tuning CNN classifiers for bone marrow cytology.

## 5 Discussion

This work explored a comprehensive and metaheuristically guided framework for integrating bone marrow cell classification with competitive and state-of-the-art deep learning architectures, as well as binarized and continuous optimization approaches. For this research, the performance gains achieved through a well-thought-out process of using a rigorous four-phase experimental design (baseline evaluation, feature selection, post-selection retraining, and hyperparameter optimization) to reduce data dimensionality, as well as dynamically adapt model parameters, were demonstrated. While prior studies have used metaheuristic techniques for either feature selection or hyperparameter tuning in isolation, they often lack an integrated pipeline capable of simultaneously handling both binary and continuous optimization challenges. This methodological gap hampers synergy between optimization stages and restricts overall model performance. The following section discusses these findings in context, elaborates on their impacts beyond medical image analysis, highlights their similarities with existing literature, and suggests possible directions for future pursuit.

Bone marrow cell images were known to be a complicated classification problem from the very beginning due to their intrinsic cytological complexity and the diversity of cell types present. The baseline results of the unoptimized models corroborated this challenge. Although impressive in performance, CNN did not outperform other architectures, such as ViT, GAN, SimCLR, or MoCo. The results indicated that applying deep learning models to high-dimensional medical image data was impossible without considering any feature refinement or parameter calibration.

The study’s methodology was significantly advanced by implementing binary metaheuristic algorithms for feature selection. Among the six algorithms evaluated, the binary variant of the Ocotillo Optimization Algorithm (bOcOA) proved to be the most successful, as it achieved the lowest classification error and selected the smallest average subset of features. This result is significant as it demonstrates bOcOA’s ability to yield high discriminative fidelity at the cost of a substantial decrease in the feature space’s dimension, thereby limiting computational overhead and avoiding overfitting. Unlike conventional filter-based or wrapper-based approaches, which often rely on rigid statistical thresholds or exhaustive combinatorics, bOcOA dynamically balances exploration and exploitation in the search space, allowing it to select compact and informative feature subsets adaptively. However, beyond numerical optimization, the selected feature subsets can also reflect clinically meaningful markers. For instance, the smaller feature sets identified by bOcOA often retained cellular morphology features associated with specific lineages or abnormalities, aiding interpretability for hematologists. This forms a basis for further studies to validate the biological relevance of these features in clinical workflows.

Compared to other binary metaheuristics, bOcOA demonstrated the lowest average error (0.4238 vs. 0.6161 in bPSO and 0.5792 in bWOA), smallest average subset size (0.3772 vs. 0.7166 in bPSO), and lowest variation in fitness, reflecting robust convergence behavior. These gains were attributed to OcOA’s feedback-informed learning, sinusoidal amplitude modulation, and convergence-aware control functions, which enable dynamic adaptation between exploration and exploitation.

The empirical gains after feature selection were also significant for all deep-learning models. Reduction in input dimensionality before model training has positively influenced the classification metrics (accuracy, precision, sensitivity, specificity, and F1-score), as these metrics have consistently improved. For example, sensitivity in CNN increased from 86.02% to 93.31%, and specificity from 86.53% to 93.62%, demonstrating an approximate 8.5% improvement in both metrics. These gains are crucial in medical diagnostics, where reducing false negatives and positives can impact treatment outcomes. Such results are consistent with those obtained from previous studies in biomedical imaging, which have demonstrated that feature selection can improve learning efficiency and generalize well even to unseen data. Moreover, the gained performance was generalizable to other model architectures, indicating that the feature selection process applies to various model architectures.

However, once the hyperparameter optimization was carried out in the second stage, it resulted in much further improvement, as was demonstrated for the CNN model. It was found that the optimizer utilizing the continuous form of OcOA performed best among all optimizers, including more established algorithms such as PSO, GWO, FA, GA, and WOA. OcOA + CNN achieved the highest post-optimization accuracy (98.24%), with sensitivity rising to 98.34% and specificity to 98.14%, compared to PSO + CNN’s 96.38% and GWO + CNN’s 95.90%. This finding also addresses a major shortcoming in previous research: the lack of optimization methods capable of maintaining convergence stability while exploring large hyperparameter spaces in high-dimensional deep learning models. This is explained by OcOA’s hybrid sinusoidal-cosine mutation and Gaussian-based refinements, which support a strong balance between global search and local exploitation. The convergence factor *K* and learning factor *L* evolve in response to search progress, ensuring more efficient exploration during early iterations and refined convergence later. This behavior prevents premature stagnation, a known issue in PSO and WOA.

**Fig 26:**
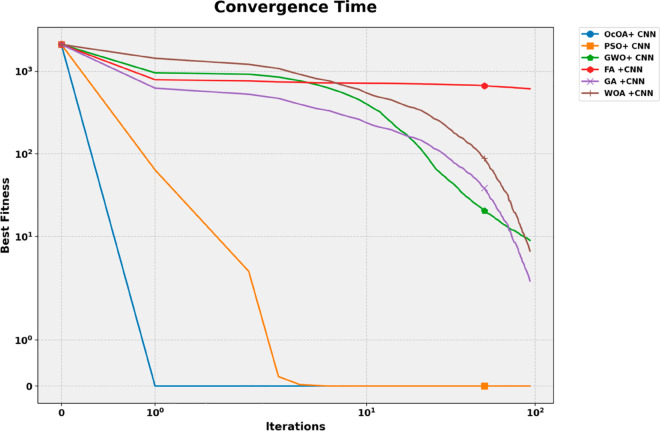
Convergence behavior of CNN optimized using different metaheuristics. OcOA demonstrates the fastest and most stable convergence, reaching minimal fitness within a few iterations.

The convergence behavior of the proposed optimizer is visualized in Fig 26, which plots the best fitness values against iteration count on a logarithmic scale. OcOA+CNN demonstrates a rapid drop in fitness—converging in fewer than 10 iterations—compared to slower decay in GWO, GA, and WOA. This faster convergence is due to OcOA’s hybrid feedback mechanisms that dynamically shift between exploration and exploitation. Mathematically, the adaptive learning factor $L=\frac{1-2\sin(\Theta )}{(1+8\cos(\Theta ){)}^{2}}$ and the convergence-sensitive factor $K=1\;-\;(\frac{\text{Gaussian}(\mu )}{A+D}{)}^{2}$ continuously update the search trajectory, enhancing convergence rate without sacrificing precision. The position update rule $\Theta (t+1)=\Theta_{t}+A\cdot D+r_{1}\cdot L$ in exploration, and $\Theta (t+1)=\text{Gaussian}(\mu )+L_{1}r_{1}\frac{A}{i\text{Train}}+F\frac{A}{o}$ in exploitation, allows the optimizer to explore early and fine-tune later aggressively. These characteristics are reflected in OcOA’s steep convergence slope and stability beyond early iterations, demonstrating superior fitness minimization efficiency compared to PSO, FA, and others.

Finally, We Compare these results with those from previous medical image classification studies in related work. The conclusion is that the combined use of metaheuristic feature selection and hyperparameter tuning is a strong paradigm for medical image classification. However, in previous works, these techniques have been used one at a time, i.e., they have been applied using PSO or GA to tune and filter-based techniques for feature reduction. Only a few have addressed the problem in a wholly integrated manner, enabling both binary and continuous metaheuristics within a single pipeline. As a result, these fragmented strategies often lack coherence and reproducibility across tasks. The unified OcOA-based pipeline presented here fills this void by enabling simultaneous optimization across multiple model configurations and learning paradigms. For this reason, the present study proposes a novel and holistic framework, which can be easily extended to other diagnostic imaging tasks (e.g., radiomics, histopathology, and digital cytology).

However, several limitations exist to such encouraging results. First, the dataset used in this study is large and diverse; however, its source is a single clinical institution. Left unanswered is the question of model generalizability to data undergoing staining with different protocols, imaging resolutions, and other factors within a clinical workflow. Future work will validate the proposed framework using data from multiple institutions to establish external validity. Second, class imbalance in certain cellular categories, although mitigated through stratified sampling, may still impact model generalization, particularly in rare cell subtypes. Advanced class balancing techniques, such as focal loss or synthetic oversampling, could further strengthen future iterations of the framework. Studying deep learning models is complex, especially when such models are used in high-risk medical applications. Without addressing specific forms of attention maps and feature importance scores, this conceptual study would benefit from the integration of explainable AI (XAI) techniques to further enhance model clarity and clinician belief.

Moreover, while the metaheuristic algorithms performed well, they are stochastic and computationally intensive. Convergence was obtained within the acceptable iteration thresholds. However, the computational load—particularly during hyperparameter tuning with OcOA—could be a limiting factor in large-scale real-time deployments. Further research may consider hybrid optimization frameworks that combine deterministic heuristics with metaheuristics to reduce runtime while maintaining performance effectiveness. Dynamic algorithm adaptation, which refers to a generalization whereby the parameters of the optimizer change in response to the feedback provided by the training process, may also offer additional improvements in efficiency and stability.

From a clinical integration perspective, this framework could be embedded into diagnostic pipelines through decision support modules linked to digital pathology systems. For example, pathologists could use automated suggestions on probable cell classifications, which they can verify or override. Integration with laboratory information systems (LIS) would enable seamless data handling and management. However, real-world deployment must contend with variations in staining quality, scanner calibration, and inter-institutional imaging protocols, all of which may affect model performance. Additionally, real-time inference on high-resolution slides requires optimized hardware or edge AI deployment, which may not be feasible in all clinical laboratories.

Other critical limitations include the interpretability of selected features. While OcOA effectively reduces dimensionality, it does not inherently explain which features are biologically meaningful. This limitation can hinder clinical adoption where interpretability is vital. Moreover, generalizing from this dataset to global patient populations remains an open challenge.

Future directions should explore the integration of attention-based architectures (e.g., vision transformers with attention map visualization) to enhance interpretability. Furthermore, deploying the model under federated learning settings across institutions could improve generalization while preserving patient privacy. Incorporating domain adaptation techniques to adjust for staining and imaging variability will also be critical for robust, cross-site deployment.

The findings of this study present strong evidence about the effectiveness of metaheuristically optimized deep learning models for classifying bone marrow cell images. Flaws: Any combination of bOcOA and OcOA results in superior performance of the models compared to state-of-the-art models, as evidenced by both predictive accuracy and diagnostic reliability. These results suggest that such frameworks could enable the functional deployment of future clinical decision support systems, ultimately facilitating faster and more scalable hematological diagnostics.

## 6 Conclusion and future work

This study presents an intelligent diagnostic framework for bone marrow cell classification, utilizing the Ocotillo Optimization Algorithm (OcOA) in feature selection and hyperparameter optimization. Demonstrate scalability in a bio-inspired form by combining binary and continuous OcOA to enhance deep learning models, such as CNNs, ViTs, and contrastive learning methods, within a streamlined, fully automated pipeline. Binary OcOA and the deep learning architecture were combined to achieve synergy, significantly improving classification accuracy, generalization, and efficiency over a framework that was a strong candidate for real-world deployment in hematological diagnostics.

For example, this work goes beyond the core performance improvements. It provides a methodologically coherent pipeline that supplements the literature’s significant gap and lack of continuous and discrete integrated optimization strategies that work consistently in these domains, demonstrating the robustness and adaptability of OcOA through a comparative analysis of some state-of-the-art metaheuristics. Showed that OcOA is not a standalone optimizer but a modular component to improve many medical imaging systems. Additionally, the framework is well for supervised and self-supervised learning paradigms and has the potential for low-label or label-scarce clinical settings.

For future work, deploying the proposed system across multiple institutional datasets will be explored to assess its generalizability in different clinical environments. Other extensions of the OcOA framework could include introducing explainable AI components to make the AI more interpretable and increase clinicians’ comfort in using the model, as well as extending the OcOA framework to support multimodal data fusion, real-time diagnostic support, and adaptive learning in evolving clinical settings. The proposed methodology has excellent potential to establish itself as the backbone of Aifuel’s hematological analysis for future generations led by AI.
